# Indicated Prevention for Children Screened in Routine Health Care: Effectiveness of a Social Skills Program on Social Anxiety and Depressive Symptoms

**DOI:** 10.1007/s10802-024-01221-w

**Published:** 2024-07-04

**Authors:** Julia Zink, Max Weniger, Patricia Theresa Porst, Cornelia Beate Siegmund, Maria McDonald, Frank Rückert, Veit Roessner, Susanne Knappe, Katja Beesdo-Baum

**Affiliations:** 1 Behavioral Epidemiology, Institute of Clinical Psychology and Psychotherapy, TUD Dresden University of Technology, Chemnitzer Straße 46, 01187 Dresden, Germany; 2 Department of Child and Adolescent Psychiatry, Faculty of Medicine, TUD Dresden University of Technology, Dresden, Germany; 3 Evangelische Hochschule Dresden (ehs), University of Applied Sciences for Social Work, Education and Nursing, Dürerstraße 25, 01307 Dresden, Germany

**Keywords:** Indicated prevention, Children, Social anxiety, Depression, Social skills

## Abstract

Social anxiety is common in childhood and potentially transitions into clinical disorders including depression. The present study aimed to examine the effectiveness of an indicated prevention program for children screened in routine care. Data came from the PROMPt project, a prospective implementation study (10/2018-09/2022) that explored a novel care chain, starting with screening with the Strengths and Difficulties Questionnaire (SDQ) as part of regular health check-ups, followed by indicated assignment and participation in a prevention program. Questionnaires assessing anxiety and depression symptoms were administered shortly after screening or before program participation (T0), six months after screening or after program participation (T1) and at a follow-up, six months after T1 (T2). Children who participated in a group cognitive-behavioral social skills program (TT; *n* = 145) were compared using mixed model analyses with non-participating children who were either screened as normal (NOR; *n* = 894) or refused program participation despite indication (NoTT; *n* = 67). TT scores improved from T0 to T1 compared to NOR and NoTT (anxiety β = -0.71 and β = -0.71, social anxiety β = -0.46 and β = -0.52, depression β = -0.52 and β = -0.73). Improvement was maintained at T2. Moderation analyses showed a trend toward greater benefit for participants with higher baseline scores. Indicated prevention can improve anxiety and depression symptoms in children identified by screening in routine care. Systematic screening and targeted prevention may positively affect mental health of children on a population level.

**Supplementary Information:**

The online version contains supplementary material available at 10.1007/s10802-024-01221-w.

Social anxiety disorder is one of the most common mental disorders with an estimated lifetime prevalence of 4–7% (Niermann et al., 2021; Stein et al., 2017) and high comorbidity with depressive disorders (Beesdo et al., 2007; Stein et al., 2017), leading to considerable suffering and severe impairments in psychosocial functioning (Kessler, 2003). Incidence of social anxiety disorder is most pronounced in childhood and early adolescence (Knappe et al., 2015; Stein et al., 2017), comorbid depressive disorders most often emerge in adolescence and adulthood (Schleider et al., 2014). In particular, social anxiety disorder has been considered a precursor to incident (i.e., secondary) depressive disorders (Beesdo et al., 2007). Further, it has been shown that full-blown disorders are usually preceded by subclinical symptoms, providing a starting point for early intervention to counteract unfavorable trajectories (Ahlen & Ghaderi, 2020).

Subclinical emotional (internalizing) problems, including anxiety and depressive symptoms, were reported in a large proportion of children (Ahlen & Ghaderi, 2020; Sterba et al., 2007). According to parent reports from the German Health Interview and Examination Survey for Children and Adolescents (KiGGS), 6.4% of 3–6 year olds and 7.9% of 7–10 year olds showed borderline emotional problems, and 11.0% of 3-6-year-olds and 10.5% of 7-10-year-olds showed borderline problems in dealing with peers, i.e., they were more likely to be alone, less popular, and teased (Hölling et al., 2007). Emotional problems were associated with immediate negative consequences in children’s daily lives, such as loneliness or school avoidance (Weeks et al., 2009). Although developmental trajectories were found to be heterogeneous, there were children whose early emotional problems persisted into late childhood and early adolescence, making them particularly vulnerable to the development of full-blown mental disorders (Ahlen & Ghaderi, 2020; Clark et al., 2007; Sterba et al., 2007) and emphasizing the need for early indicated prevention.

Focusing on the association of social anxiety and depression, interpersonal factors have been suggested as mediators (Epkins & Heckler, 2011; Starr et al., 2014). Specifically, low sociability and interpersonal hypersensitivity (Starr et al., 2014) as well as loneliness (Ebesutani et al., 2015) and peer victimization (Biggs et al., 2010) have been found to be associated with depressive symptoms in social anxiety. Considering the social skills deficit vulnerability model by Segrin and colleagues (2016), poor social skills are associated with reduced available social support, leading to increased psychological distress like depression or loneliness. That is, social anxiety and depression might be countered preventively at an early age by strengthening social skills and helping children overcome avoidance behaviors, thus fostering positive social experiences, especially with peers (Epkins & Heckler, 2011; Hetrick et al., 2015; Moreno-Peral et al., 2017; Neil & Christensen, 2009; Teubert & Pinquart, 2011).

One way to intervene early is through indicated prevention, targeting those with subclinical symptoms (Donovan & Spence, 2000). In Germany, the indicated prevention program “Mutig werden mit Til Tiger” (“Becoming Brave with Til Tiger”, Til Tiger program) (Ahrens-Eipper et al., 2010) has been conceptualized for fearful and shy children aged 5–10 years. This cognitive-behavioral group program aims to provide new strategies for dealing with fearful situations, reduce avoidance behaviors, and increase self-esteem. It focuses on practical exercises improving social skills. The group setting also provides an opportunity for positive peer experiences. The program has been positively evaluated using a waiting list control design: Compared to the waiting control group, the intervention group showed significantly decreased social anxiety, increased social competence and self-esteem (Ahrens-Eipper et al., 2010). Further evaluation of this program, however, is still pending, particular pertaining to its effects in routine health care settings.

In general, small to moderate effect sizes in terms of symptom reduction can be expected for indicated prevention of anxiety and depression in children and adolescents (Hetrick et al., 2015; Moreno-Peral et al., 2017; Neil & Christensen, 2009; Teubert & Pinquart, 2011). A recent meta-analysis regarding the FRIENDS program showed that anxiety and depression symptoms were effectively reduced in participants compared to controls, with effect sizes of Cohen’s d = -0.20 and d = -0.24 at post-intervention and d = -0.20 and d = -0.21 at follow-up (Fisak et al., 2023). Van Starrenburg and colleagues (2017) examined the effects of the Coping Cat program offered as indicated prevention for primary school children and showed a reduction in anxiety symptoms with an effect size of d = -0.66 at follow-up. Moreover, looking at meta-analyses, the study by Teubert and Pinquart (2011), which included different anxiety prevention programs for children and adolescents up to age 18, showed small effects for indicated/selective prevention in reducing anxiety symptoms (Hedges g = 0.32 at posttest and g = 0.23 at follow-up). Likewise, the meta-analysis by Rasing and colleagues (2017) on indicated/selective cognitive-behavioral prevention of anxiety and depression in adolescents found small effects in reducing symptoms, but the effects were only temporary. Ooi and colleagues (2022) conducted a meta-analysis to examine the efficacy of selective/indicated interventions for preschool children with increased levels of behavioral inhibition. They found moderate effects in reducing anxiety symptoms. However, there is also evidence that prevention can reduce not only the severity of symptoms, but also the incidence of full-blown disorders (Moreno-Peral et al., 2017; Ooi et al., 2022; Stockings et al., 2016).

Most of the mentioned studies on prevention efficacy were randomized controlled trials (RCT) as a high standard method to investigate effects. Nevertheless, the investigation of the effectiveness under routine conditions also seems important in light of the challenges to indicated prevention: First, the identification of children with subthreshold symptoms, and second, the recommendation of and participation in an appropriate prevention program. Although the “prevention law” in Germany supports health promotion and prevention, only a small proportion of the population utilizes such programs (Bauer et al., 2020) despite the option for reimbursement by many health-insurance companies. Even among children and adolescents with anxiety or mood disorders, only about one-third and one-half, respectively, seek or receive treatment (Merikangas et al., 2010; Niermann et al., 2021). Several reasons for this intervention gap have been discussed, including that parents do not recognize emotional problems or underestimate their negative effects (Reardon et al., 2017). To address this, early screening for symptoms could be beneficial, e.g. in the pediatric setting as an established and accepted care structure (Schmidtke et al., 2018). It is therefore important to investigate the effects of targeted allocation to low-threshold, indicated prevention programs in a naturalistic setting.

Against this background, the present study used data from an indicated prevention implementation study in which children were screened for emotional problems using the Strengths and Difficulties Questionnaire (SDQ; emotional problems scale) (Goodman, 1997) during their routine health check-up at their pediatrician’s office. If indicated children were recommended to participate in the Til Tiger program. The present study aimed to examine the following research questions (1) whether program participation improves social anxiety, overall anxiety, and depressive symptoms in children screened positive in routine health care, i.e. program effectiveness, and (2) whether initial levels of social anxiety, anxiety, and depressive symptoms moderate the effectiveness of the prevention program.

We hypothesized that the participating children’s anxiety and depression symptoms would improve relative to their scores before the prevention program and approach the scores of children who were screened as normal. We also hypothesized that the scores of the non-participating children, who were recommended to participate in the Til Tiger program but did not do so, would not change. The latter was expected, as epidemiologic studies showed both stable as well as waxing and waning patterns among individuals with anxiety symptoms (Becker et al., 2015).

We further aimed to examine whether the initial level of anxiety and depressive symptoms moderates the effectiveness of the prevention program. This is important to determine the symptom level at which this prevention program works best, still works, or does not work. We examined this question exploratively because previous findings on initial symptom severity in indicated prevention have been inconsistent. Studies showed that higher initial symptom severity was associated with either worse (Dadds et al., 1999; Roberts et al., 2003) or better outcomes (Gillham et al., 2001), or was not associated with outcome at all (Brière et al., 2014; Makover et al., 2019). In addition, since prevention programs differ in several aspects (e.g., content, duration, age of participants, parent sessions), they may be difficult to compare in this regard.

## Methods

### Sample and Procedures

The current study used data from the PROMPt project, an indicated prevention implementation study (10/2018–09/2022) conducted in Dresden, Germany (Weniger et al., 2022). The PROMPt study protocol as well as its amendments were reviewed and accepted by the ethics committee of the TUD Dresden University of Technology (TUD: EK200052019) and a confirmatory vote of the State Chamber of Physicians of Saxony (AZ: EK-allg-18/19 − 1) was obtained. The PROMPt study was conducted in accordance with the ethical standards of the 1975 Declaration of Helsinki (as amended), the German (European) data protection regulations and the guidelines of Good Clinical Practice. PROMPt aimed to identify children at increased risk for developing mental health problems early in routine health care and to allocate them to an established indicated prevention program. For this, parents were asked to fill in the SDQ (Goodman, 1997, 1999) during the regular health check-ups (“U9”, children usually aged 5–6 years; “U10”, 7–8 years; “U11” 9–10 years). Pediatricians then provided feedback to parents based on SDQ scores on the emotional problems scale or conduct problems scale (disruptive behavior problems; not part of this manuscript), as well as their clinical expertise on whether the child could benefit from an indicated prevention program. Children with emotional problems received a recommendation for the program “Becoming brave with Til Tiger” (TT) (Ahrens-Eipper et al., 2010). If children had high (abnormal) SDQ scores for emotional (> 6) or disruptive behavior problems (> 5) and clinically relevant problems according to the pediatrician’s expertise, parents were recommended to contact counseling or therapeutic institutions (not part of this manuscript).

In case of interest in participation in a prevention program, parents were to contact the study team directly. If a prevention program was recommended but the family did not contact the study team within 3–4 weeks, the study team attempted to contact the family up to five times to determine whether they intended to participate, provided consent to contact was available. Written consent to be contacted and to participate in the study was obtained at the screening by the pediatrician. In addition to this allocation route, other routes of accession were also monitored, e.g., self-assignment, recommendation by friends or acquaintances. All families were invited to an initial interview conducted by psychologists to determine if the child would benefit from the program and inclusion criteria were fulfilled. Participation in the prevention program was only possible if written consent of the custodian and the verbal consent of the child were available, obtained at the initial interview, and no diagnosis of an emotional/disruptive behavior disorder according to ICD-10 was reported within the last 6 months, as well as no unstable medication, no ongoing psychotherapy, and no acute risk to self or others.

A flowchart of the sample is provided in the Online Resource 1. A total of *n* = 3,231 children were screened at the pediatrician and *n* = 139 children entered the project via other access routes, resulting in *n* = 3,370 screened children. Of these, informed consent was not available for *n* = 406 children, these were not considered further, resulting in *n* = 2,964. Of these, *n* = 1,936 children were classified as normal (NOR), meaning they were not recommended for a prevention program or other intervention by the pediatrician or study team. *N* = 416 children were recommended for the Til Tiger program by the pediatrician or the study team because of emotional problems. Of these, *n* = 145 children participated in the Til Tiger prevention program (TT) and *n* = 271 children did not participate in the program despite recommendation (NoTT). The remaining *n* = 612 children were excluded as not relevant to the current analyses (see Online Resource 1). As many children dropped out from screening to the other measurement time points, children only were retained for the current analyses when data from at least one measurement time point besides screening were available. This resulted in *n* = 143 for TT, *n* = 894 for NOR and *n* = 67 for NoTT, summing up to *n* = 1,104.

Because children were included in the study via screening by the pediatricians, it occurred that the same child was present twice but as distinct cases in the data set, for example, when the family attended regular health examinations with the child at ages 8 and 10 years. In the current data set, there were three children with two data entries each. One child was categorized once as TT and once as NoTT, the other two were each categorized twice as NOR.

### Prevention Program

The Til Tiger program is a cognitive-behavioral group program conceptualized for fearful and shy children aged 5–10 years. The program consists of eleven sessions of one hour each. In our study, the program was conducted weekly, and the procedure was as follows: The first two sessions were one-on-one sessions where the child was introduced to the trainer and Til Tiger, a hand puppet portraying a tiger who was as similarly anxious and fearful as the child and suggested that both should learn to become brave. The following nine sessions were group sessions, each covering a different main topic, e.g., doing something in front of the group or rejecting something. All group sessions were structured similarly: First, the exercise of the previous session was discussed, then the main topic was introduced and practiced in role play. For each topic, behavioral strategies were taught, such as paying attention to the appropriate volume, tone of voice, and eye contact when saying “no”. Finally, a short form of progressive muscle relaxation was performed. The children were then asked to practice the new skills until the next session. The content was communicated to the parents verbally and with an information sheet after each session. The program was the same for all age groups (children were grouped +/- 1 year), but the difficulty of the exercises could be adapted to the children’s level by adding sample situations from their everyday life. The overall aim of the program was to increase the children’s self-confidence, help them cope with stress, and build and strengthen social skills. With the help of Til Tiger, the children were taught that there are peers who feel the same way (“I’m not different”), that they have rights (“I can talk to others/ refuse something/ make legitimate demands/ defend myself”). Moreover, children were encouraged that becoming brave and acting confidently in different social situations is something they can achieve, together with Til Tiger (Ahrens-Eipper et al., 2010).

The prevention program was conducted by certified trainers, who were psychologists or psychology students with a bachelor’s degree trained by the program developer in a two-day course, and took place at the TUD Dresden University of Technology. Free supervision by a child and adolescent psychotherapist was available when needed. Since the program took place under the conditions of the Corona pandemic, the program was adapted to the hygiene regulations (e.g. 3–5 children instead of 6 children per group). Program participation costed 130€ per child, with the amount often covered completely or in part by health insurance usually under the condition of 80% course participation.

### Data Collection

Data were collected at four measurement time points, namely at screening by the pediatrician (or by the study team in the case of other access routes); at T0, shortly after screening or just prior to program participation; at T1, approximately six months after screening and after program participation; and at T2, approximately twelve months after screening (i.e., 6 months after T1 or program participation). At screening, families completed the SDQ and a brief project-specific questionnaire at the pediatrician’s office, which included socio-demographic data. Families who did not participate in a prevention program received T0, T1 and T2 assessments as online questionnaires. Here, T0 questionnaires were sent as soon as possible after screening and all questionnaires were parent reports. Families who participated in a prevention program completed T0 and T1 via tablets before and after the program at the study site, respectively, and received T2 as an online questionnaire. Here, parent reports as well as children self-reports were assessed. Parents of participating children (TT) received 10€ for each assessment (T0 - T2), the other families could win family games in a raffle. A detailed overview of the data collection can be found elsewhere (Weniger et al., 2022). The mean time gap in days between screening and T0 was M = 51.9 (SD = 34.3) days for NOR, M = 121.9 (SD = 72.1) for TT and M = 82.2 (SD = 61.8) for NoTT, differing significantly between the groups (Chi2(2) = 167.74, *p* <.001). The mean time gap between T0 and T1 was M = 162.7 (SD = 37.3) for NOR, M = 104.8 (SD = 31.3) for TT and M = 123.7 (SD = 45.8) for NoTT, differing significantly between the groups (Chi2(2) = 218.01, *p* <.001). The mean time gap between T0 and T2 was M = 337.2 (SD = 36.1) for NOR, M = 287.6 (SD = 54.0) for TT and M = 281.0 (SD = 52.7) for NoTT, differing significantly between the groups (Chi2(2) = 100.36, *p* <.001).

### Measures

Socio-demographic information provided by parents were assessed at screening via a project-specific questionnaire. (Biological) sex of the child was assessed as male or female, gender identity was not assessed. Nationality was assessed as German or non-German (other). In addition, the SDQ with impact supplement (Goodman, 1997, 1999) was performed at screening, with the emotional problems scale being relevant for indicated assignment to the Til Tiger program. The questions referred to the past six months and were answered on a 3-point Likert scale (0 “not true”, 1 “somewhat true”, 2 “certainly true”). The impact supplement asked about perceived difficulties, distress, social impairment and burden to the family (Goodman, 1999). For descriptive statistics, sum scores were computed for the emotional problems scale as well as the total score (sum of the scales emotional problems, conduct problems, hyperactivity /inattention, peer relationship problems). From the impact supplement, the distress and burden items were reported and an impact score was calculated using the 0012 scoring proposed by Goodman (1999). A maximum of two missing values were allowed for each scale, with the missing values imputed by the mean of the respective subscale. The emotional problems scale and total score were categorized in normal (scale: 0–3; total: 0–13), borderline (scale: 4–6; total: 14–16) and abnormal (scale: 7–10; total: 17–40) scores (Weniger et al., 2022). Emotional problems scale cutoffs were project specific: Thresholds for abnormal scores were raised to reach more children with potential indication for prevention. The German parent version of the SDQ showed good homogeneity (α = 0.82 for the total score and α = 0.58 − 0.76 for the scale scores) in a representative norming sample and correlated strongly with other established measures (Klasen et al., 2003).

Child anxiety and depression were assessed by parental reports at T0, T1 and T2, using the Screen for Child Anxiety Related Emotional Disorders (SCARED) (Birmaher et al., 1999) and Center for Epidemiologic Studies Depression Scale for Children (CES-DC) (Radloff, 1977), respectively. The SCARED was answered on a 3-point Likert scale (0 “not true or hardly ever true”, 1 “sometimes true”, 2 “true or often true”), a sum was calculated for the subscales and the total score, with missing values imputed by the mean of the respective subscale. We allowed a maximum of 30% missing values, so in two cases the total score was not calculated. Otherwise, there was only one case with one missing value that was imputed. The SCARED has been shown to be internally consistent in the analyses sample (total score α = 0.90 − 0.91; social anxiety scale score α = 0.89; depending on the measurement time point) as well as in a sample of outpatient children and adolescents and their parents (α = 0.90), with parent-child correlations at moderate levels, especially for younger children (Birmaher et al., 1999). The total score discriminated between children with and without anxiety disorders, indicating good discriminant validity (Birmaher et al., 1999).

The CES-DC refers to the past week and was answered on a 4-point Likert-scale (0 “not at all”, 1 “a little”, 2 “some”, 3 “a lot”). A sum was calculated for the total score, with a maximum of 20% missing values being allowed (Radloff, 1977), so in one case the total score was not calculated, all other cases had no missing values. The CES-DC has been shown to be internally consistent in the analyses sample (α = 0.83 − 0.86 depending on the measurement time point) as well as in a sample of 7- to 10-year-old children as a parent version (α = 0.82) (Barkmann et al., 2008). Self-report scores have also been correlated with diagnoses of major depression and dysthymia (Barkmann et al., 2008; Fendrich et al., 1990). The inter-rater agreement between the child’s self-report and the parent’s report was found to be low, so the parent’s report can only be considered as an approximation (Barkmann et al., 2008). However, information on the reliability and validity of parental reports for children under age 7 is still pending.

### Description and Handling of Missing Data

As the prevention program in the PROMPt project was applied using a naturalistic (pragmatic), observational study design, subgroup sample sizes and dropout rates varied widely. There were high dropout rates from screening to the subsequent measurement time points in the NOR and especially NoTT groups, but not in the TT group, which can be explained by the study design. The screening during the regular health check-up was probably perceived by the families as a common health service, so that dropouts might be explained by a generally low motivation for longer-term study participation. The even higher dropout rate for NoTT may be due to the fact that this group consisted of families who decided not to participate in the prevention program despite the recommendations, which was often accompanied by a decision not to participate in the study at all. For TT, data were collected locally, linked to program participation, so there was little dropout here. Due to this dropout pattern, we decided to include only children in our analyses for whom anxiety or depression scores were available at least at one measurement time point. An overview of available data and a comparison of sociodemographic data between included and excluded children is provided in the Online Resources 2 and 3.

In conclusion, the missing data pattern was not assumed to be missing completely at random (MCAR) or not missing at random (NMAR), but rather missing at random (MAR), since the missingness was assumed to depend on the group but not on the level of anxiety or depression. A comparison of socio and clinical characteristics of completer (data on CES and SCARED available at all measurement times) and non-completer (data on CES and SCARED available at least at one but not all measurement time points) is provided in the Online Resource 4. Little’s test for the assumption of MCAR was not significant, both without (Chi2 distance (329) = 320.35, *p* =.623) and with group as an auxiliary variable (Chi2 distance (987) = 479.92, *p* = 1.00).

To account for missing data on depression and anxiety scores, we used mixed model analyses with previously multiple-imputed data. Mixed model analyses have been shown to produce robust results even without prior multiple imputation (MI) (Twisk et al., 2013), but baseline values are needed to determine whether initial symptom severity moderates the effectiveness of the prevention program. In those models, all subjects with missing T0 (= baseline) data would be excluded. This would severely reduce the analysis sample and lead to bias. For this, we applied MI for all mixed model analyses. In defining the MI model, we included CES and SCARED scores at all measurement time points, SDQ emotional problems scale score, sex and age of child, and group as auxiliary variable. The seed was randomly set to 4286 and the number of imputations to 15 before analyses. As the number of imputations is not trivial, especially when the number of missing values is high, we conducted sensitivity analyses with up to 100 imputations. There were no meaningful differences in the results, so for reasons of efficiency, we maintained the previously determined number of imputations (White et al., 2011). For comparison purposes, we also calculated the analyses related to the first research question without the prior use of multiple imputation.

### Statistical Analyses

Statistical analyses were conducted using STATA 17.0 (StataCorp, 2021). Descriptive statistics (number of participants, n; percent, %; mean values, M; standard deviation, SD) were provided separately for each group (NOR, TT and NoTT) and compared using the chi-square test or the Kruskal-Wallis test. If significant, effect sizes were reported (Cramér’s V, epsilon squared ε^2^) (Tomczak & Tomczak, 2014). When necessary, Bonferroni-corrected pairwise Dunn comparisons were calculated for post hoc analyses. Nonparametric tests were used for the sample description analyses because the assumptions for parametric tests were violated.

All the following analyses were performed for SCARED total score, SCARED social anxiety scale score, and CES-DC depression score. Social anxiety was specifically studied as this was the primary focus of the prevention program. To address the first research question, whether overall anxiety, social anxiety, and depressive symptoms improved in TT compared to NOR and NoTT, linear multilevel models were run on the interaction of time point and group, with random intercepts for each participant. To improve comparability, the dependent variables were standardized by the pooled standard deviation of the respective measure at T0. This allows the coefficients to be interpreted as effect sizes using Cohen’s specification (≥ 0.20 small, ≥ 0.50 medium, ≥ 0.80 large effect) (Cohen, 1988). In the depression model, the number of participants was *n* = 1,094 instead of *n* = 1,104 because depression scores were not available at any time point for *n* = 10 children in the analysis sample (see Online Resource 2). Time point and group were dummy coded, using T0 and NOR or NoTT as reference. Post hoc estimates, i.e., Bonferroni-corrected pairwise comparisons between groups at each time point, were calculated to disentangle the interaction effect if significant. For comparison, models were run without and with prior multiple imputation. In addition, explorative moderation analyses by sex of child and age of child were calculated. For this purpose, age of child was categorized using the median (Md = 6 years) into younger (6 years or younger) and older (7 years or older) children.

To address the second research question, whether initial levels of anxiety and depression moderate the effectiveness of the prevention program, subgroups were formed based on the median at T0 to handle multicollinearity of the continuous T0 score with the group variable. That is, within the groups TT and NoTT, participants were assigned to either a high (> median) or low ( < = median) group for each outcome measure at T0 (anxiety, social anxiety, and depression). Linear multilevel models were run on the interaction of time point, group, and median group with random intercepts for each participant. Dependent variables were standardized, and time point, group, and median group were coded as a dummy, using T0, TT, and low as reference. These models were only run with multiple imputations due to missing T0 data, i.e., missing values in the median group variable (as described above). When the three-way interaction effect was significant, separate models were run for each group (TT and NoTT) to disentangle the effect. In addition, predictive margins were displayed for ease of interpretation. All analyses were adjusted for child age, child sex and time in days between measurements. Robust standard errors were calculated. The alpha level was set a priori at α = 0.05.

## Results

### Sample Characteristics and Descriptive Statistics

Table 1 shows the socio-demographic and clinical characteristics separately for each group, i.e., NOR, TT and NoTT. There were differences between the groups in child sex (Chi2(2) = 6.53, *p* =.038, V = 0.08), with TT having more boys and NoTT having more girls, and in siblings (Chi2(2) = 8.97, *p* =.011, V = 0.10), with TT having more only children and NOR having more siblings. In terms of SDQ scores at screening, the groups differed on the emotional problems scale (Chi2(2) = 438.85, *p* <.001, ε^2^ = 0.40) and the total score (Chi2(2) = 291.75, *p* <.001, ε^2^ = 0.27). Pairwise comparisons showed that NOR had lower scores than TT and NoTT (each *p* <.001), whereas TT and NoTT did not differ significantly (emotional problems scale score: *p* =.979, total score: *p* =.390). On the SDQ impact supplement, the groups differed significantly on perceived difficulties (Chi2(6) = 355.86, *p* <.001, V = 0.41), impact score (Chi2(2) = 385.45, *p* <.001, ε^2^ = 0.37) and burden rating (Chi2(6) = 352.94, *p* <.001, V = 0.41), with TT reporting higher levels more frequently than NoTT and both more frequently than NOR.

**Table 1:** Socio-demographic and clinical characteristics

	NOR			TT			NoTT			Chi2(df)	*p*
	*n* = 894			*n* = 143			*n* = 67				
	*n*	M(SD) %		*n*	M(SD) %		*n*	M(SD) %			
**Age of mother**											
mean age [M(SD)]	735	37.8(4.7)		106	37.4(5.0)		41	37.7(4.9)		2.33(2)	.312
20–29 years	23	3.1		1	0.9		1	2.4		9.20(6)	.163
30–39 years	449	61.1		71	67.0		24	58.5			
40–49 years	256	34.8		30	28.3		15	36.6			
50–59 years	7	1.0		4	3.8		1	2.4			
**Age of father**											
mean age [M(SD)]	709	40.6(6.1)		99	39.4(4.6)		38	39.3(5.5)		4.87(2)	.088
20–29 years	13	1.8		1	1.0		0	0.0		7.85(8)	.448
30–39 years	313	44.2		52	52.5		20	52.6			
40–49 years	330	46.5		44	44.4		17	44.7			
50–59 years	49	6.9		2	2.0		1	2.6			
60–69 years	4	0.6		0	0.0		0	0.0			
**Age of child**											
mean age [M(SD)]	893	6.7(1.9)		141	6.6(1.8)		66	6.5(1.9)		0.62(2)	.734
4 years	18	2.0		4	2.8		3	4.6		6.19(8)	.626
5–6 years	433	48.5		73	51.8		32	48.5			
7–8 years	237	26.5		40	28.4		20	30.3			
9–10 years	200	22.4		24	17.0		11	16.7			
11 years	5	0.6		0	0.0		0	0.0			
**Sex of child**											
male	410	46.0		78	54.6		24	36.4		**6.53(2)**	**.038**
female	481	54.0		65	45.5		42	63.6			
**Nationality of mother**											
German	716	97.2		102	97.1		38	92.7		2.63(2)	.268
other	21	2.9		3	2.9		3	7.3			
**Nationality of father**											
German	692	97.3		99	99.0		38	100.0		2.03(2)	.363
other	19	2.7		1	1.0		0	0.0			
**Nationality of child**											
German	721	98.8		103	99.0		39	100.0		0.53(2)	.766
other	9	1.2		1	1.0		0	0.0			
**Institution**											
kindergarten/preschool	436	50.5		70	49.0		32	52.5		0.22(2)	.894
school	428	49.5		73	51.1		29	47.5			
**Siblings**											
at least one	624	83.3		104	72.7		38	80.9		**8.97(2)**	**.011**
none	125	16.7		39	27.3		9	19.2			
**Living situation**											
single parent	70	9.5		13	12.3		6	14.0		3.21(4)	.523
permanent partnership/married	637	86.7		89	84.0		35	86.1			
other	28	3.8		4	3.8		0	0.0			
**Houshold net income after tax**											
less than 1000€	7	1.1		2	2.1		0	0.0		7.03(8)	.533
1000–2000€	74	11.1		11	11.5		3	8.6			
2000–3000€	104	15.6		18	18.8		10	28.6			
3000–4000€	253	37.8		32	33.3		14	40.0			
more than 4000€	231	34.5		33	34.4		8	22.9			
**Mother’s highest educational qualification**											
without educational qualifications	1	0.1		0	0.0		0	0.0		2.42(10)	.992
lower secondary education	19	2.6		2	1.9		1	2.4			
secondary school certificate	197	26.8		29	27.4		9	22.0			
higher education entrance qualification	156	21.2		22	20.8		10	24.4			
graduate degree	349	47.5		50	47.2		21	51.2			
other	13	1.8		3	2.8		0	0.0			
**Father’s highest educational qualification**											
without educational qualifications	2	0.3		2	2.0		0	0.0		9.71(10)	.467
lower secondary education	31	4.4		6	6.0		1	2.6			
secondary school certificate	216	30.3		24	24.0		13	34.2			
higher education entrance qualification	109	15.3		18	18.0		5	13.2			
graduate degree	336	47.1		48	48.0		17	44.7			
other	19	2.7		2	2.0		2	5.3			
**SDQ (Screening)**											
**emotional problems scale score [M(SD)]**	890	1.2(1.3)		138	5.2(1.9)		66	4.8(1.7)		**438.85(2)**	**<.001**
normal	854	96.0		23	16.7		9	13.6		**697.72(4)**	**<.001**
borderline	30	3.4		89	64.5		47	71.2			
abnormal	6	0.7		26	18.8		10	15.2			
**total score [M(SD)]**	890	5.7(3.6)		138	13.4(5.1)		66	11.9(4.7)		**291.75(2)**	**<.001**
normal	868	97.5		77	55.8		43	65.2		**305.76(4)**	**<.001**
borderline	18	2.0		24	17.4		12	18.2			
abnormal	4	0.5		37	26.8		11	16.7			
**SDQ impact supplement**											
**perceived difficulties**											
no	555	64.6		12	9.2		12	19.1		**355.86(6)**	**<.001**
minor	280	32.6		54	41.2		35	55.6			
definite	20	2.3		56	42.8		14	22.2			
severe	4	0.5		9	6.9		2	3.2			
**impact score [M(SD)]**	858	0.2(0.6)		132	2.1(1.7)		63	1.2(1.5)		**385.45(2)**	**<.001**
**burden rating**											
no	663	77.6		18	13.7		21	67.1		**352.9(6)**	**<.001**
minor	173	20.3		58	44.3		29	24.8			
definite	17	2.0		48	36.6		10	7.2			
severe	1	0.1		7	5.3		2	1.0			

Note. NOR: children who were not recommended to participate in a prevention program because they were assessed as normal; TT: children who participated in the prevention program “Mutig werden mit Til Tiger” (Becoming brave with Til Tiger), i.e. attended at least one session; NoTT: children who were recommended to participate in the Til Tiger program but did not do so; all variables except siblings were assessed at the screening timepoint; if institution information was missing at screening, information from timepoint T0 was used; sibling were assessed at timepoint T0; The *n* = 4 children in the TT age group “4 years” were 5 years old at start of the program due to the time gap between screening and program start/T0; Cut-offs for the emotional problems scale score of the SDQ were project-specific: normal 0-3, borderline 4-6, abnormal 7-10; Cut-offs for the SDQ total score were: normal 0-13, borderline 14-16, abnormal 17-40;The SDQ impact supplement has more missing data because it was on the back of the SDQ questionnaire and thus likely overlooked; n: number of cases; M: mean; SD: standard deviation; Chi2: Chi Square Test or Kruskal-Wallis-Test; df: degrees of freedom; bold indicates statistical significance, *p* <.05

The TT children attended an average of M = 9.88 (SD = 1.63) sessions of the prevention program, which corresponds to 89.8%. *N* = 6 children, 4.2%, dropped out prematurely (mean number of sessions participated: M = 4.50, SD = 1.87, min = 2, max = 7).

### Effectiveness of Program Participation on Anxiety, Social Anxiety, and Depression

**Fig. 1:**
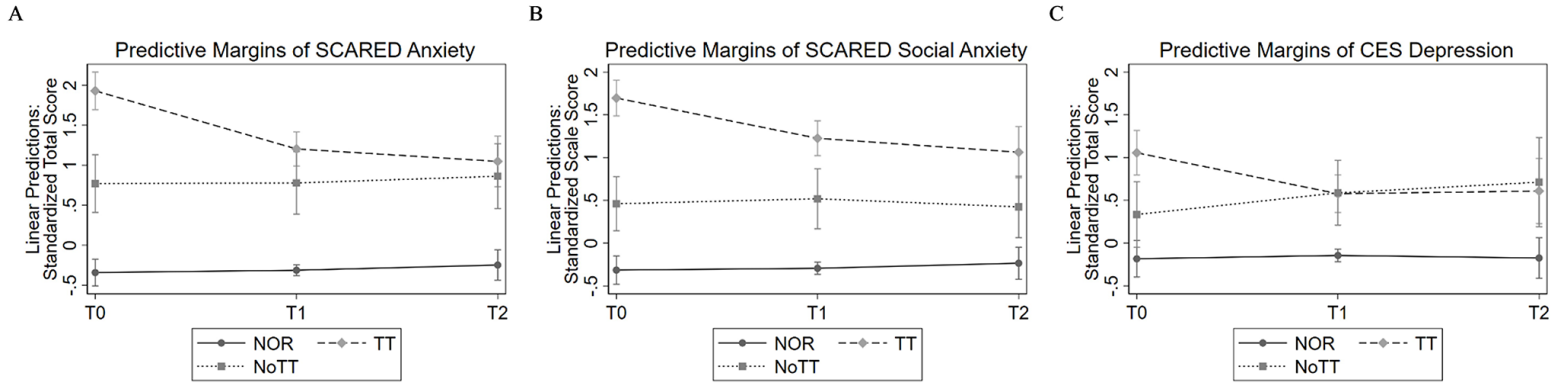
Predictive margins based on multilevel regression models analyzing the effect of group and time point on (A) anxiety, (B) social anxiety, and (C) depression. The dependet variables were standardized. T0, measurement time point before program participation; T1, first measurement time point after program participation, (approximately 3–5 months after T0); T2, second measurement time point after program participation, (approximately 9–11 months after T0); NOR: children who were not recommended to participate in a prevention program because they were assessed as normal; TT: children who participated in the prevention program; NoTT: children who were recommended to participate in the program but did not do so; all models were adjusted for age of child, sex of child and time in days between measurements; Error bars indicate 95% confidence interval

The results of the multilevel models are presented in Table 2; Fig. 1. The pattern of results was similar for anxiety, social anxiety, and depression: Each model showed an interaction between group and time point, with only TT but not NoTT interacting with time point when the reference was NOR, and only TT but not NOR interacting with time point when the reference was NoTT. This means that the reduction from T0 to T1 or from T0 to T2 was greater in TT than in NOR and NoTT. This was observed for anxiety, social anxiety, and depression. The standardized coefficients, which represent effect sizes, are shown in Table 2.

**Table 2:** Multilevel regression models for anxiety, social anxiety, and depression on the interaction of group and time point using multiply imputed data

		SCARED Anxiety				SCARED Social Anxiety				CES-DC Depression			
		β	SE	95%CI	*p*	β	SE	95%CI	*p*	β	SE	95%CI	*p*
**Model 1: NOR as reference**													
Measurement Time Point	T0	reference											
	T1	0.03	0.09	-0.14–0.20	.741	0.02	0.08	-0.14–0.19	.803	0.04	0.11	-0.18–0.26	.727
	T2	0.09	0.17	-0.24–0.43	.581	0.08	0.17	-0.25–0.41	.630	0.01	0.22	-0.42–0.44	.968
Group	NOR	reference											
	TT	**2.27**	**0.12**	**2.04–2.51**	**<.001**	**2.01**	**0.10**	**1.81–2.21**	**<.001**	**1.24**	**0.12**	**1.00–1.48**	**<.001**
	NoTT	**1.11**	**0.18**	**0.76–1.46**	**<.001**	**0.78**	**0.16**	**0.47–1.08**	**<.001**	**0.52**	**0.19**	**0.15–0.89**	**.006**
Time Point x Group	T1 x TT	**-0.75**	**0.12**	**-0.98**– **-0.53**	**<.001**	**-0.49**	**0.09**	**-0.67**– **-0.31**	**<.001**	**-0.52**	**0.14**	**-0.80**– **-0.23**	**<.001**
	T1 x NoTT	-0.02	0.16	-0.34–0.29	.896	0.04	0.16	-0.29–0.36	.822	0.22	0.23	-0.24–0.67	.352
	T2 x TT	**-0.98**	**0.12**	**-1.21–0.74**	**<.001**	**-0.71**	**0.11**	**-0.93**– **-0.50**	**<.001**	**-0.46**	**0.14**	**-0.74**– **-0.17**	**.002**
	T2 x NoTT	0.00	0.18	-0.35–0.35	.994	-0.12	0.14	-0.39–0.16	.405	0.37	0.28	-0.19–0.92	.192
	age of child	**0.03**	**0.02**	**0.00–0.06**	**.030**	-0.01	0.01	-0.04–0.01	.327	**0.04**	**0.01**	**0.01–0.07**	**.004**
	sex of child	-0.07	0.06	-0.19–0.05	.237	-0.08	0.06	-0.19–0.04	.184	0.04	0.06	-0.07–0.15	.497
	time gap in days	0.00	0.00	0.00–0.00	.439	0.00	0.00	0.00–0.00	.568	0.00	0.00	0.00–0.00	.769
	intercept	**-0.47**	**0.11**	**-0.69**– **-0.24**	**<.001**	-0.12	0.11	-0.34–0.10	.299	**-0.53**	**0.11**	**-0.75**– **-0.32**	**<.001**
**Model 2: NotTT as reference**													
Measurement Time Point	T0	reference											
	T1	0.01	0.17	-0.32–0.34	.965	0.06	0.17	-0.28–0.39	.734	0.25	0.24	-0.21–0.72	.285
	T2	0.09	0.22	-0.34–0.52	.673	-0.04	0.19	-0.40–0.33	.844	0.38	0.34	-0.28–1.04	.261
Group	NoTT	reference											
	NOR	**-1.11**	**0.18**	**-1.46**– **-0.76**	**<.001**	**-0.78**	**0.16**	**-1.08**– **-0.47**	**<.001**	**-0.52**	**0.19**	**-0.89**– **-0.15**	**.006**
	TT	**1.16**	**0.21**	**0.75–1.56**	**<.001**	**1.24**	**0.18**	**0.89–1.58**	**<.001**	**0.72**	**0.21**	**0.30–1.14**	**.001**
Time Point x Group	T1 x NOR	0.02	0.16	-0.29–0.34	.896	-0.04	0.16	-0.36–0.29	.822	-0.22	0.23	-0.67–0.24	.352
	T1 x TT	**-0.73**	**0.19**	**-1.11**– **-0.36**	**<.001**	**-0.53**	**0.18**	**-0.88**– **-0.17**	**.004**	**-0.73**	**0.26**	**-1.25**– **-0.22**	**.005**
	T2 x NOR	0.00	0.18	-0.35–0.35	.994	0.12	0.14	-0.16–0.39	.405	-0.37	0.28	-0.92–0.19	.192
	T2 x TT	**-0.97**	**0.21**	**-1.38**– **-0.57**	**<.001**	**-0.60**	**0.17**	**-0.93**– **-0.27**	**<.001**	**-0.82**	**0.31**	**-1.43**– **-0.21**	**.008**
	age of child	**0.03**	**0.02**	**0.00–0.06**	**.030**	-0.01	0.01	-0.04–0.01	.327	**0.04**	**0.01**	**0.01–0.07**	**.004**
	sex of child	-0.07	0.06	-0.19–0.05	.237	-0.08	0.06	-0.19–0.04	.184	0.04	0.06	-0.07–0.15	.497
	time gap in days	0.00	0.00	0.00–0.00	.439	0.00	0.00	0.00–0.00	.568	0.00	0.00	0.00–0.00	.769
	intercept	**0.64**	**0.21**	**0.24–1.05**	**.002**	**0.66**	**0.19**	**0.28–1.04**	**.001**	-0.02	0.22	-0.45–0.42	.943

Note. T0, measurement time point before program participation; T1, first measurement time point after program participation, (approximately 3-5 months after T0); T2, second measurement time point after program participation, (approximately 9-11 months after T0); NOR: children who were not recommended to participate in a prevention program because they were assessed as normal; TT: children who participated in the prevention program “Mutig werden mit Til Tiger” (Becoming brave with Til Tiger), i.e. attended at least one session; NoTT: children who were recommended to participate in the Til Tiger program but did not do so; group and time point were coded as a dummy, using T0 and NOR or NoTT as the reference; all models were adjusted for age of child, sex of child (0 =  female, 1 =  male) and time in days between measurements (time gap); CI, confidence interval; SE, standard error; bold indicates statistical significance, p <.05

Pairwise comparisons at each time point showed the following results: At T0, TT had higher scores than NOR (anxiety: 95%CI: 1.98–2.56; social anxiety: 95%CI: 1.77–2.25; depression: 95%CI: 0.94–1.54) and than NoTT (anxiety: 95%CI: 0.67–1.65; social anxiety: 95%CI: 0.81–1.66; depression: 95%CI: 0.21–1.24). NoTT had higher scores than NOR (anxiety: 95%CI: 0.69−1.54; social anxiety: 95%CI: 0.40–1.15; depression: 95%CI: 0.07–0.97).

At T1, scores of TT and NoTT did not differ for anxiety (95%CI: -0.12–0.97) and depression (95%CI: -0.55–0.52). For social anxiety, scores of TT were higher than NoTT (95%CI: 0.21–1.20). For all measures at T1, TT had higher scores than NOR (anxiety: 95%CI: 1.24–1.79; social anxiety: 95%CI: 1.26–1.78; depression: 95%CI: 0.44–1.00), and NoTT had higher scores than NOR (anxiety: 95%CI: 0.61–1.57; social anxiety: 95%CI: 0.38–1.25; depression: 95%CI: 0.26–1.21).

The same pattern was found at T2: TT and NoTT scores did not differ at T2 for anxiety (95%CI: -0.37–0.74) and depression (95%CI: -0.77–0.56), but TT had higher social anxiety scores than NoTT (95%CI: 0.15–1.13). TT had higher scores than NOR (anxiety: 95%CI: 0.98–1.61; social anxiety: 95%CI: 1.00–1.60; depression: 95%CI: 0.43–1.14), and NoTT had higher scores than NOR (anxiety: 95%CI: 0.64–1.58; social anxiety: 95%CI: 0.25–1.07; depression: 95%CI: 0.31–1.46).

Models using data without prior multiple imputation yielded similar results and can be found in the Online Resource 5. Explorative moderation analyses by sex of child and age of child showed no significant three-way interaction effects (sex of child: each *p* > = .054; age of child: each *p* > = .114). Detailed results of the models are provided in the Online Resource 6 and 7.

### Moderation by Initial Symptom Severity

**Fig. 2:**
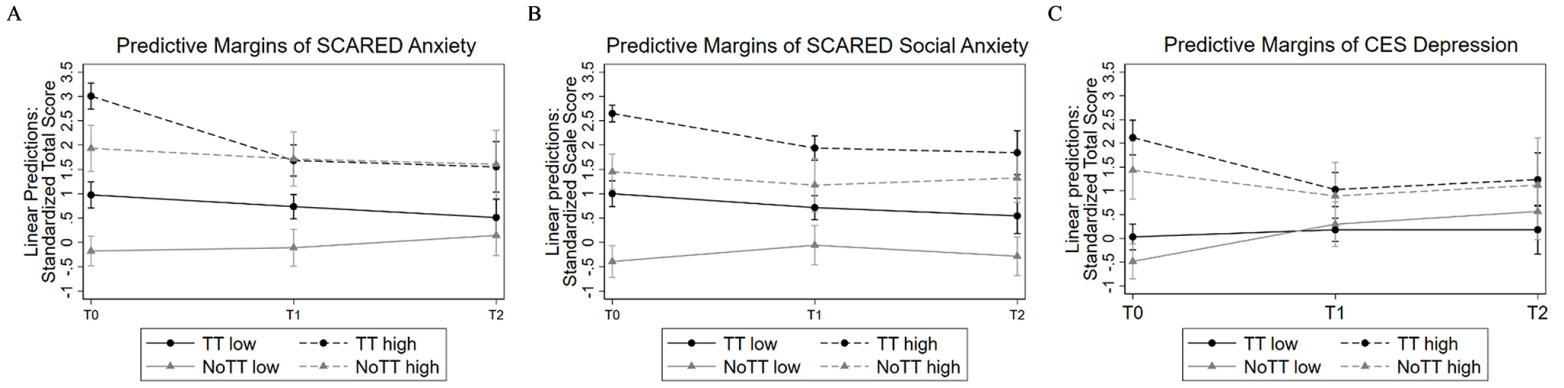
Predictive margins based on multilevel regression models analyzing the interaction of group, median group, and time point on (**A**) anxiety, (**B**) social anxiety, and (**C**) depression. The models were run with multiply imputed data and the dependet variables were standardized. Children were grouped into either the high (> median) or low ( < = median) mediangroup; T0, measurement time point before program participation; T1, first measurement time point after program participation, (approximately 3–5 months after T0); T2, second measurement time point after program participation, (approximately 9–11 months after T0); TT: children who participated in the prevention program; NoTT: children who were recommended to participate in the program but did not do so; all models were adjusted for age of child, sex of child and time in days between measurements; Error bars indicate 95% confidence interval

The multilevel regression models revealed a significant three-way interaction of time point (T0, T1, T2), group (TT, NoTT), and median group (low, high) for anxiety, but not for social anxiety and depression (each *p* >.600). For anxiety, there was an interaction regarding T1(β = 0.80, SE = 0.38, 95%CI: 0.05–1.54, *p* =.037), but not T2 (β = 0.34, SE = 0.42, 95%CI: -0.49–1.16, *p* =.423). That is, the difference from T0 to T1 in TT high compared to TT low was greater than the difference across time in NoTT. Predictive margins are displayed in Fig. 2, detailed results of the models are provided in the Online Resource 8. Disentangling the three-way interaction found for anxiety, separate regression models for TT and NoTT revealed, that there was a time point and median group interaction for TT but not NoTT. In TT, the difference from T0 to T1 and for T0 to T2 was greater in the high group than in the low group (T1: β = -1.08, SE = 0.19, 95%CI: -1.46– -0.70, *p* <.001; T2: β = -0.99, SE = 0.21, 95%CI: -1.41– -0.57, *p* <.001). For NoTT, the differences between T0 and T1 or T2 were not different in the low and high groups (T1: β = -0.26, SE = 0.32, 95%CI: -0.88–0.36, *p* =.410; T2: β = -0.66, SE = 0.36, 95%CI: -1.36–0.04, *p* =.063).

Looking at Fig. 2, the interaction effect for anxiety is clearly visible. But also for depression, it seems that the scores improved more in the TT high group than in the TT low group. However, this interaction effect was not significant, as the worsening from T0 toT1 for NoTT low was similar in magnitude to the improvement for TT high.

## Discussion

The aim of the present study was to examine whether elevated symptoms of overall anxiety, social anxiety, and depression in children identified through screening in routine care would be reduced by participation in an indicated prevention program. In addition, we aimed to determine whether the initial severity of symptoms moderated the effectiveness of the program. Our main findings were that symptoms of anxiety, social anxiety, and depression improved in children who participated in the program, and that this improvement was sustained over the medium term. We further found that the effectiveness tended to be stronger in children with higher symptom levels at baseline. However, contrary to our hypothesis, the level of symptoms of the participating children did not approach that of the children classified as normal, but that of the children who did not participate despite program recommendation.

The Til Tiger program is conceptualized for anxious and shy children aged 5 to 10 years and aims to teach social skills and to reduce avoidance behaviors. Evaluating this program in the context of indicated prevention using data from a nonrandomized pragmatic implementation study, we found that participation improved symptoms of anxiety, social anxiety, and depression, supporting and extending previous findings (Ahrens-Eipper et al., 2010). More specifically, the scores of children who participated (TT) improved compared to children who were assessed as normal (NOR), whereas the scores of children who did not participate despite being recommended for participation (NoTT) did not change in this comparison. This effect was found immediately after the end of the program and was maintained for six months, indicating at least medium-term stability.

However, the anxiety and depression scores of TT did not normalize to the level of NOR, but reached the less impaired level of NoTT; for social anxiety the TT scores were still above the level of both other groups. In this regard it is important to note that the initial symptom severity differed between the three groups. While the low NOR scores are intuitive, the difference between TT and NoTT needs to be discussed. On both the SDQ and the specific anxiety and depression questionnaires, TT had descriptively higher initial scores than NoTT. The differences were significant on the specific questionnaires, but not on the SDQ. It can be assumed that the SDQ, as a shorter screening instrument, measured the severity of emotional problems less sensitively, which may have led to the lack of significance. The difference between TT and NoTT can be explained by the naturalistic, observational character of the study: Those who rated lower symptom severity were more likely to feel less burdened resulting in lower subjective need for participation in a prevention program. This aligns with the most frequently reported reasons for rejection: no need, no time or problem has improved on its own (Weniger et al., 2024). This suggests that screening at the pediatrician’s office was sensitive and tended to recommend a program to families even if they themselves did not feel it was necessary. Although several studies have demonstrated the good psychometric properties of the SDQ, including its high sensitivity and specificity for screening for mental disorders in children (Klasen et al., 2003; Lehmann et al., 2014), further research on the use of the SDQ for prevention recommendations is suggested. In particular, evidence for screening for anxiety in younger children (7 years of age or younger) seems to be limited (US Preventive Services Task Force et al., 2022), suggesting that further research is needed, including consideration of other screening instruments for internalizing (emotional) problems or social anxiety (Garcia-Lopez et al., 2015).

Nevertheless, a reduction in symptoms to the level of NoTT could still be considered a meaningful change as NoTT has felt less impaired and burdened. As these are subclinical conditions, reducing symptoms to this level may still reduce the risk of later mental disorders. This could not be tested with our data, but the findings provide a starting point for further research. In addition, it would be useful for future research to ask not only about symptom severity, but also about the extent to which the prevention program had an impact on coping with anxiety in everyday life, e.g., whether safety and avoidance behaviors were reduced. On the other hand, the still higher social anxiety scores could also indicate that this program may not be sufficient or, in the context of the Corona pandemic, may not have reached its full potential due to adaptations (including fewer opportunities to practice learned skills at home, fewer children in a group). The difference in effectiveness between anxiety and social anxiety suggests that the program also had a positive effect on other anxiety symptoms, probably even more than on social anxiety. Hence, the effectiveness of the program in preventing social anxiety as compared to overall anxiety or emotional problems should be further investigated. Especially since it is likely that the children in the TT group were not exclusively affected by social anxiety symptoms: On the one hand, the program recommendations were based on the SDQ emotional problems scale score, i.e., not on a specific measure of social anxiety. On the other hand, the indication interview was used to determine whether the child would benefit from the Til Tiger program, with other subthreshold anxieties not being a reason for exclusion. In addition, the waxing and waning of subclinical symptoms indicates that the risk of symptom progression remains elevated (Becker et al., 2015).

Nonetheless, the effect sizes we found were within the range expected for prevention. In a meta-analysis of the effectiveness of prevention in children and adolescents in German-speaking countries, Beelmann and colleagues (2014) found an effect size of d = 0.41 for indicated prevention and d = 0.21 for prevention of emotional problems. A review of school-based prevention of anxiety found effects ranging from 0.20 to 0.76 for indicated prevention when a significant prevention effect was reported (Neil & Christensen, 2009). Small effect sizes were also found for anxiety prevention (not focused on children or indicated for prevention) in the meta-analysis by Moreno-Peral and colleagues (2017). That is, although the scores did not approach those of children classified as normal, the improvements in participants’ anxiety and depression were within the expected range for indicated prevention programs in children. In addition, the improvements can be considered clinically meaningful because the symptom levels approached those of NoTT, who were less impaired and burdened at screening than TT. Although the improvement in TT cannot be clearly attributed to an effect of the prevention program due to the different baseline scores, an improvement due to regression to the mean alone is unlikely. The NoTT scores were also significantly higher than the NOR scores and therefore should have shown a tendency to regress to the mean. However, this was not found in the data. Apart from that, additional booster sessions may be beneficial to further improve program effectiveness (Garcia-Lopez et al., 2023), which should be investigated in future research.

Another aspect of our study was to examine whether the effectiveness of the prevention program was moderated by the initial symptom severity. Previous studies of other indicated prevention programs have yielded mixed results - however, these are difficult to compare due to differences in programs and study designs (Brière et al., 2014; Gillham et al., 2001; Makover et al., 2019). In the present study, there was a tendency for children with higher baseline scores to benefit more from the prevention program, at least for anxiety and depression symptoms. This moderation was not found for social anxiety in particular, but the overall effectiveness of the prevention program was lower here. Although indicated prevention implies that only children with subthreshold emotional symptoms were recommended to participate, this trend suggests that the eligibility threshold could be further raised to target those children who would benefit most from the prevention and who are at higher risk of symptom progression due to high initial scores. However, as the trend was not found for social anxiety, this would risk excluding less socially anxious children who would still benefit. More targeted screening and grouping prior to program participation (e.g., by symptom severity) may be useful, but more research is needed to make a confident recommendation. It would be necessary to evaluate whether the resource requirements associated with a more specific allocation would be justified by improved program effectiveness, or whether a simpler screening process would be more feasible. Further, when interpreting this, it is important to keep in mind that due to the observational character of our study, the baseline scores of the median divided groups were different. That is, TT high had higher baseline scores than NoTT high, which were more comparable to TT low. Therefore, it cannot be excluded that the greater change in TT scores was driven by other factors, such as spontaneous remissions or waxing and waning of symptoms. However, the stability of the NoTT scores argues against this possibility.

In summary, our results showed that indicated prevention was effective under routine conditions. The Til Tiger program, which focused on promoting social skills and overcoming avoidance behavior in social situations, was shown to reduce (social) anxiety and depressive symptoms in children at increased risk for developing a full-blown disorder, and this seemed to be stable at least in the medium term. The routine conditions showed that families did not enroll in the program until symptoms reached a certain level of severity or accompanying personal burden, even if the pediatrician recommended it. Combined with the observed tendency for children with higher baseline scores to benefit most from the program, this suggests that indicated assignment is important and that the program should not be offered universally. Screening by the pediatrician seems to be a good opportunity for indicated referral, which, together with self-selection by families, leads to a participant group that benefits from the prevention program.

There were some limitations. In terms of generalizability to the entire spectrum of the general population, it should be noted that families with higher education and socioeconomic status were more likely to participate, which is a common selection bias in epidemiologic studies (Enzenbach et al., 2019). However, in the present sample, no differences were found between the groups studied with regard to these variables, so selection bias should not be critical to the research questions. Otherwise, the sample can be considered representative for Germany (urban areas), but no conclusions can be drawn for other cultures. Our results need to be interpreted as program effectiveness under ecologically valid conditions rather than efficacy, which would have required an RCT. Further, there were a lot of missing values throughout the measurement time points, especially in the families that did not participate in the prevention program. This limitation was addressed by closely examining the structure of missing values and using elaborated methods to estimate them (mixed model analysis, multiple imputation). The results were comparable with and without prior multiple imputation, suggesting that the results were reliable. The time intervals between measurements differed between groups, but this did not affect the analyses. More critically, the baseline values differed between groups, so the interpretation of the improvement in the participants cannot be clearly attributed to the prevention program. However, the stability of the NoTT group argues against the effect of regression to the mean in the TT group. We used only parent reports to determine the anxiety and depression symptoms in the children. The correlation between parent report and child self-report was found to be moderate for the questionnaires used (Barkmann et al., 2008; Birmaher et al., 1999), so parent report can be considered an approximation. For future studies, it may be useful to include self-report and/or additional third-party measures. Although all interested families were invited to an initial interview that included asking about current and past ICD-10 diagnoses, this did not include a diagnostic interview, so we cannot rule out the presence of clinical disorders in the participating children. In addition, the SCARED items were developed using DSM-IV as basis and thus may not reflect current DSM-5 criteria, which, however, have hardly changed regarding anxiety disorders. As almost all the prevention programs and measurements were conducted under Corona pandemic conditions, the effect of the pandemic cannot be evaluated. It is likely that children were generally more distressed during this time and may have had difficulty fully practicing learned strategies at home during contact restrictions (Leopoldina, 2021). Further, we did not assess treatment integrity, however, all providers were given a clearly structured manual, were intensively trained by the developer of the program, and were regularly offered free supervision by a psychological psychotherapist. Last, we did not assess social skills directly, so we cannot conclude if improvement in social skills or other aspects of the prevention program (e.g., potential increase in self-confidence, stress management) contributed to decreases in anxiety and depression symptoms.

There were also strengths to be highlighted. The observational design of this implementation study in the general population allowed the effectiveness of the prevention program to be tested under ecologically valid conditions and adding to previous effectiveness reports on the prevention program from a waiting list control study (Ahrens-Eipper et al., 2010). Recruitment of the sample through screening at regular health check-ups provided a representative sample for the Dresden area, which allowed generalization to other (urban) regions in Germany. Another strength was the longitudinal design, which allowed us to examine changes or stability of the effectiveness of the prevention program over a six-month period after program participation.

In conclusion, an indicated prevention program, “Becoming Brave with Til Tiger”, has been shown to improve symptoms of (social) anxiety and depression in children screened in routine health care, possibly counteracting the development of full-blown emotional disorders. The program teaches children to overcome fears at an early stage and to explore favorable strategies for dealing with different social situations, which seems to be particularly important in view of the various social challenges and changes children face at school. An extended offer of the prevention program and follow-up measures therefore seems warranted. In order for children to receive the maximum benefit from the program, consideration should be given to early identification and targeted referral to the program. Future major research is needed to examine, whether the implementation of routine screening on a large scale and targeted allocation to effective prevention programs could impact public mental health by a decreased incidence of mental disorders in children and adolescents.

## Electronic Supplementary Material

Below is the link to the electronic supplementary material.

Supplementary Material 1

## Data Availability

The data that support the findings of this study are available from the senior author upon reasonable request.
