# Supplementary material for: Indicated Prevention for Children Screened in Routine Health Care: Effectiveness of a Social Skills Program on Social Anxiety and Depressive Symptoms
Source: Res Child Adolesc Psychopathol. 2024 Jul 4;52(10):1515–31. doi: 10.1007/s10802-024-01221-w (PMC11461602; doi:10.1007/s10802-024-01221-w)
Supplement: Supplementary file 1 — Supplementary Material 1 [file 10802_2024_1221_MOESM1_ESM.docx]

**Supplementary Material**

**Children screened by the pediatrician: n = 3,231**

**Children entered the project via other access routes n = 139**

**Totally screened children**

**n = 3,370**

**Available screened children**

**n = 2,964**

**Excluded due to lack of informed consent: n = 406**

**Excluded as not relevant to the current study: n = 612**

- Not fulfilling the inclusion criteria for program participation: n = 16*
- Indication for and/or participation in another prevention program (“Ein Stressbewältigungstraining für Trotzköpfe und Zornteufel”): n = 523
- Abnormal, meaning too symptomatic for prevention according to pediatrician, study team, or when information about their recommendation was not available, SDQ: n = 69
- No status available due to missing SDQ and pediatrician‘s report: n = 4

**Children relevant for the current study: n = 2,352**

- TT: n = 145
- NOR: n = 1,936
- NoTT: n = 271

**Excluded because data in the relevant variables (anxiety/depression) were not available at any measurement time point: n=1,248**

- TT: n = 2
- NOR: n = 1,042
- NoTT: n = 204

**Analysis sample: n= 1,104**

- TT: n = 143
- NOR: n = 894
- NoTT: n = 67

*Online Resource 1.* Flowchart of the analysis sample. n, number of participants; “Children screened by the pediatrician” defined as: The study team received at least one material from the screening folder the family filled in at the pediatrician, i.e., informed consent or SDQ or project specific questionnaire or pediatrician’s report. When comparing the flowchart with the more detailed flowchart by Weniger et al. (2024), it should be noted that the children via other access routes were included at different stages in the flowcharts, making it more difficult to cross-check the numbers. NOR: children who were not recommended to participate in a prevention program because they were assessed as normal; TT: children who participated in the prevention program "Mutig werden mit Til Tiger" (Becoming brave with Til Tiger), i.e., attended at least one session; NoTT: children who were recommended to participate in the Til Tiger program but did not do so.

*They were not included in the NoTT because of important differences: despite interest, they could not participate in the prevention program because of exclusion criteria (mostly ongoing psychotherapy). In NoTT, there was generally no interest in participating in the prevention program.

| Online Resource 2. Overview of available data | | | | | | | | |
| --- | --- | --- | --- | --- | --- | --- | --- | --- |
|  | NOR | |  | TT | |  | NoTT | |
|  | n=1,936 | |  | n=145 | |  | n=271 | |
|  | n | % |  | n | % |  | n | % |
| **SDQ screening total score available** | 1,927 | 99.5 |  | 140 | 96.6 |  | 269 | 99.3 |
| **SDQ screening emotional problem scale score available** | 1,928 | 99.6 |  | 140 | 96.6 |  | 269 | 99.3 |
|  |  |  |  |  |  |  |  |  |
| **Sociodemographic information available** | 1,458 | 75.3 |  | 106 | 73.1 |  | 174 | 64.2 |
|  |  |  |  |  |  |  |  |  |
| **CES total score available** |  |  |  |  |  |  |  |  |
| T0 | 730 | 37.7 |  | 141 | 97.2 |  | 45 | 16.6 |
| T1 | 645 | 33.3 |  | 134 | 92.4 |  | 38 | 14.0 |
| T2 | 600 | 31.0 |  | 106 | 73.1 |  | 31 | 11.4 |
|  |  |  |  |  |  |  |  |  |
| T0&T1 | 545 | 28.2 |  | 132 | 91.0 |  | 25 | 9.2 |
| T0&T2 | 502 | 25.9 |  | 106 | 73.1 |  | 21 | 7.7 |
| T1&T2 | 486 | 25.1 |  | 101 | 69.7 |  | 19 | 7.0 |
|  |  |  |  |  |  |  |  |  |
| at least one measurement time point | 885 | 45.7 |  | 143 | 98.6 |  | 66 | 24.4 |
| at least two measurement time points | 647 | 33.4 |  | 137 | 94.5 |  | 31 | 11.4 |
| all measurement time points | 443 | 22.9 |  | 101 | 69.7 |  | 17 | 6.3 |
|  |  |  |  |  |  |  |  |  |
| **SCARED total score/social anxiety scale available** |  |  |  |  |  |  |  |  |
| T0 | 737 | 38.1 |  | 141 | 97.2 |  | 46 | 17.0 |
| T1 | 650 | 33.6 |  | 134 | 92.4 |  | 38 | 14.0 |
| T2 | 607 | 31.4 |  | 106 | 73.1 |  | 31 | 11.4 |
|  |  |  |  |  |  |  |  |  |
| T0&T1 | 549 | 28.4 |  | 132 | 91.0 |  | 25 | 9.2 |
| T0&T2 | 505 | 26.1 |  | 106 | 73.1 |  | 21 | 7.7 |
| T1&T2 | 491 | 25.4 |  | 101 | 69.7 |  | 19 | 7.0 |
|  |  |  |  |  |  |  |  |  |
| at least one measurement time point | 894 | 46.2 |  | 143 | 98.6 |  | 67 | 24.7 |
| at least two measurement time points | 655 | 33.8 |  | 137 | 94.5 |  | 30 | 11.1 |
| all measurement time points | 445 | 23.0 |  | 101 | 69.7 |  | 17 | 6.3 |
| *Note.* Overview of available data for all cases relevant to the current study. NOR: children who were not recommended to participate in a prevention program because they were assessed as normal; TT: children who participated in the prevention program "Mutig werden mit Til Tiger" (Becoming brave with Til Tiger), i.e. attended at least one session; NoTT: children who were recommended to participate in the Til Tiger program but did not do so; SDQ: Strenghts and Difficulties Questionnaire; CES: Center for Epidemiologic Studies Depression Scale for Children; SCARED: Screen for Child Anxiety Related Emotional Disorders; Due to high dropout rates from screening to the other measurement time points, only cases that had completed the CES or SCARED at at least one measurement time point were included in the study. | | | | | | | | |
|  |  |  |  |  |  |  |  |  |
|  |  |  |  |  |  |  |  |  |
|  |  |  |  |  |  |  |  |  |
|  |  |  |  |  |  |  |  |  |

| Online Resource 3. Socio-demographic and clinical characteristics: Comparing excluded and included children | | | | | | | | | | |
| --- | --- | --- | --- | --- | --- | --- | --- | --- | --- | --- |
|  | Excluded | | |  | Included | | |  |  |  |
|  | n=1,248 | | |  | n=1,104 | | |  |  |  |
|  | n | M(SD) % | |  | n | M(SD) % | |  | t(df) Chi2(df) | p |
| **Group** |  |  |  |  |  |  |  |  |  |  |
| NOR | 1,042 | 83.5 | |  | 894 | 81.0 | |  | **209.65(2)** | **<.001** |
| TT | 2 | 0.2 | |  | 143 | 13.0 | |  |  |  |
| NoTT | 204 | 16.4 | |  | 67 | 6.1 | |  |  |  |
|  |  |  |  |  |  |  |  |  |  |  |
| **Age of mother** |  |  | |  |  |  | |  |  |  |
| mean age [M(SD), t(df)] | 829 | 37.1(4.8) | |  | 882 | 37.7(4.8) | |  | **-2.77(1709)** | **.006** |
|  |  |  | |  |  |  | |  |  |  |
| 20-29 years | 28 | 3.4 | |  | 25 | 2.8 | |  | 7.55(3) | .056 |
| 30-39 years | 558 | 67.3 | |  | 544 | 61.7 | |  |  |  |
| 40-49 years | 233 | 28.1 | |  | 301 | 34.1 | |  |  |  |
| 50-59 years | 10 | 1.2 | |  | 12 | 1.4 | |  |  |  |
|  |  |  |  |  |  |  |  |  |  |  |
| **Age of father** |  |  | |  |  |  | |  |  |  |
| mean age [M(SD), t(df)] | 781 | 39.6(5.6) | |  | 847 | 40.4(5.9) | |  | **-2.80(1625)** | **.005** |
|  |  |  | |  |  |  | |  |  |  |
| 20-29 years | 11 | 1.4 | |  | 14 | 1.7 | |  | **9.54(4)** | **.049** |
| 30-39 years | 402 | 51.5 | |  | 385 | 45.5 | |  |  |  |
| 40-49 years | 319 | 40.9 | |  | 391 | 46.2 | |  |  |  |
| 50-59 years | 49 | 6.3 | |  | 52 | 6.2 | |  |  |  |
| 60-69 years | 0 | 0.0 | |  | 4 | 0.5 | |  |  |  |
|  |  |  |  |  |  |  |  |  |  |  |
| **Age of child** |  |  | |  |  |  | |  |  |  |
| mean age [M(SD), t(df)] | 1,236 | 6.8(2.0) | |  | 1,100 | 6.7(1.9) | |  | 0.78(2334) | .438 |
|  |  |  |  |  |  |  |  |  |  |  |
| 3-4 years | 40 | 3.2 | |  | 24 | 2.3 | |  | 5.91(4) | .206 |
| 5-6 years | 578 | 46.8 | |  | 538 | 48.9 | |  |  |  |
| 7-8 years | 332 | 26.9 | |  | 297 | 27.0 | |  |  |  |
| 9-10 years | 272 | 22.0 | |  | 235 | 21.4 | |  |  |  |
| 11 years | 14 | 1.1 | |  | 5 | 0.5 | |  |  |  |
|  |  |  |  |  |  |  |  |  |  |  |
| **Sex of child** |  |  |  |  |  |  |  |  |  |  |
| male | 546 | 45.6 | |  | 512 | 46.6 | |  | 0.20(1) | .655 |
| female | 651 | 54.4 | |  | 588 | 53.5 | |  |  |  |
|  |  |  |  |  |  |  |  |  |  |  |
| **Nationality of mother** |  |  |  |  |  |  |  |  |  |  |
| German | 800 | 95.9 | |  | 856 | 96.9 | |  | 1.30(1) | .254 |
| other | 34 | 4.1 | |  | 27 | 3.1 | |  |  |  |
| **Nationality of father** |  |  |  |  |  |  |  |  |  |  |
| German | 751 | 96.0 | |  | 829 | 97.6 | |  | 3.48(1) | .062 |
| other | 31 | 4.0 | |  | 20 | 2.4 | |  |  |  |
| **Nationality of child** |  |  |  |  |  |  |  |  |  |  |
| German | 818 | 99.2 | |  | 863 | 98.9 | |  | 0.38(1) | .539 |
| other | 7 | 0.9 | |  | 10 | 1.2 | |  |  |  |
|  |  |  |  |  |  |  |  |  |  |  |
| **Institution** |  |  |  |  |  |  |  |  |  |  |
| kindergarten/preschool | 448 | 52.8 | |  | 538 | 50.4 | |  | 1.08(1) | .298 |
| school | 401 | 47.2 | |  | 530 | 49.6 | |  |  |  |
|  |  |  |  |  |  |  |  |  |  |  |
| **Siblings** |  |  |  |  |  |  |  |  |  |  |
| at least one | 38 | 76.0 | |  | 766 | 81.6 | |  | 0.97(1) | .325 |
| none | 12 | 24.0 | |  | 173 | 18.4 | |  |  |  |
|  |  |  |  |  |  |  |  |  |  |  |
| **Living situation** |  |  |  |  |  |  |  |  |  |  |
| single parent | 118 | 14.2 | |  | 89 | 10.1 | |  | **8.99(2)** | **.011** |
| permanent partnership/married | 675 | 80.9 | |  | 761 | 86.3 | |  |  |  |
| other | 41 | 4.9 | |  | 32 | 3.6 | |  |  |  |
|  |  |  |  |  |  |  |  |  |  |  |
| **Houshold net income after tax** |  |  |  |  |  |  |  |  |  |  |
| less than 1000€ | 5 | 0.7 | |  | 9 | 1.1 | |  | **16.30(4)** | **.003** |
| 1000-2000€ | 116 | 16.5 | |  | 88 | 11.0 | |  |  |  |
| 2000-3000€ | 141 | 20.1 | |  | 132 | 16.5 | |  |  |  |
| 3000-4000€ | 241 | 34.3 | |  | 299 | 37.4 | |  |  |  |
| more than 4000€ | 200 | 28.5 | |  | 272 | 34.0 | |  |  |  |
|  |  |  |  |  |  |  |  |  |  |  |
| **Mother's highest educational qualification** |  |  |  |  |  |  |  |  |  |  |
| without educational qualifications | 3 | 0.4 | |  | 1 | 0.1 | |  | **47.60(5)** | **<.001** |
| lower secondary education | 31 | 3.8 | |  | 22 | 2.5 | |  |  |  |
| secondary school certificate | 330 | 40.2 | |  | 235 | 26.6 | |  |  |  |
| higher education entrance qualification | 168 | 20.5 | |  | 188 | 21.3 | |  |  |  |
| graduate degree | 276 | 33.6 | |  | 420 | 47.6 | |  |  |  |
| other | 13 | 1.6 | |  | 16 | 1.8 | |  |  |  |
|  |  |  |  |  |  |  |  |  |  |  |
| **Father's highest educational qualification** |  |  |  |  |  |  |  |  |  |  |
| without educational qualifications | 5 | 0.7 | |  | 4 | 0.5 | |  | **31.47(5)** | **<.001** |
| lower secondary education | 47 | 6.2 | |  | 38 | 4.5 | |  |  |  |
| secondary school certificate | 292 | 38.6 | |  | 253 | 29.7 | |  |  |  |
| higher education entrance qualification | 140 | 18.5 | |  | 132 | 15.5 | |  |  |  |
| graduate degree | 257 | 34.0 | |  | 401 | 47.1 | |  |  |  |
| other | 16 | 2.1 | |  | 23 | 2.7 | |  |  |  |
|  |  |  |  |  |  |  |  |  |  |  |
| **SDQ (Screening)** |  |  | |  |  |  | |  |  |  |
| **emotional problems scale score [M(SD), t(df)]** | 1,243 | 1.7(1.9) | |  | 1,094 | 1.9(2.1) | |  | **-2.50(2224.12)** | **.013** |
|  |  |  |  |  |  |  |  |  |  |  |
| normal | 1,023 | 82.3 | |  | 886 | 81.0 | |  | 5.65(2) | .059 |
| borderline | 193 | 15.5 | |  | 166 | 15.2 | |  |  |  |
| abnormal | 27 | 2.2 | |  | 42 | 3.8 | |  |  |  |
|  |  |  |  |  |  |  |  |  |  |  |
| **total score [M(SD), t(df)]** | 1,242 | 7.1(4.9) | |  | 1,094 | 7.1(4.8) | |  | 0.02(2334) | .986 |
|  |  |  |  |  |  |  |  |  |  |  |
| normal | 1,120 | 90.2 | |  | 988 | 90.3 | |  | 0.54(2) | .765 |
| borderline | 68 | 5.5 | |  | 54 | 4.9 | |  |  |  |
| abnormal | 54 | 4.4 | |  | 52 | 4.8 | |  |  |  |
| *Note*. Included: data on CES or SCARED available at least one measurement time point (=analysis sample); Excluded: data on CES and SCARED not available at any measurement time point; NOR: children who were not recommended to participate in a prevention program because they were assessed as normal; TT: children who participated in the prevention program "Mutig werden mit Til Tiger" (Becoming brave with Til Tiger), i.e. attended at least one session; NoTT: children who were recommended to participate in the Til Tiger program but did not do so; all variables except siblings were assessed at the screening timepoint; if institution information was missing at screening, information from timepoint t0 was used; sibling were assessed at timepoint t0; n: number of cases; M: mean; SD: standard deviation; Chi2: Chi Square Test (for categorical variables); t: (Welch's) t-test (for continuous variables); bold prints indicate statistical significance, p<.05 | | | | | | | | | | |

| Online Resource 4. Socio-demographic and clinical characteristics: Comparing non-completer and completer | | | | | | | | | | |
| --- | --- | --- | --- | --- | --- | --- | --- | --- | --- | --- |
|  | Non-Completer | | |  | Completer | | |  |  |  |
|  | n=543 | | |  | n=561 | | |  |  |  |
|  | n | M(SD) % | |  | n | M(SD) % | |  | t(df) Chi2(df) | p |
| **Group** |  |  |  |  |  |  |  |  |  |  |
| NOR | 451 | 83.1 | |  | 443 | 79.0 | |  | **40.39(2)** | **<.001** |
| TT | 42 | 7.7 | |  | 101 | 18.0 | |  |  |  |
| NoTT | 50 | 9.2 | |  | 17 | 3.0 | |  |  |  |
|  |  |  | |  |  |  | |  |  |  |
| **Age of mother** |  |  | |  |  |  | |  |  |  |
| mean age [M(SD), t(df)] | 413 | 37.5(4.9) | |  | 469 | 38.0(4.7) | |  | -1.63(880) | .103 |
|  |  |  |  |  |  |  |  |  |  |  |
| 20-29 years | 18 | 4.4 | |  | 7 | 1.5 | |  | **8.78(3)** | **.032** |
| 30-39 years | 254 | 61.5 | |  | 290 | 61.8 | |  |  |  |
| 40-49 years | 138 | 33.4 | |  | 163 | 34.8 | |  |  |  |
| 50-59 years | 3 | 0.7 | |  | 9 | 1.9 | |  |  |  |
|  |  |  |  |  |  |  |  |  |  |  |
| **Age of father** |  |  | |  |  |  | |  |  |  |
| mean age [M(SD), t(df)] | 395 | 40.1(5.5) | |  | 451 | 40.8(6.2) | |  | -1.64(844) | .101 |
|  |  |  |  |  |  |  |  |  |  |  |
| 20-29 years | 6 | 1.5 | |  | 8 | 1.8 | |  | 6.23(4) | .183 |
| 30-39 years | 190 | 48.1 | |  | 195 | 43.2 | |  |  |  |
| 40-49 years | 179 | 45.3 | |  | 212 | 47.0 | |  |  |  |
| 50-59 years | 20 | 5.1 | |  | 32 | 7.1 | |  |  |  |
| 60-69 years | 0 | 0.0 | |  | 4 | 0.9 | |  |  |  |
|  |  |  |  |  |  |  |  |  |  |  |
| **Age of child** |  |  | |  |  |  | |  |  |  |
| mean age [M(SD), t(df)] | 541 | 6.6(1.9) | |  | 559 | 6.7(1.9) | |  | -0.81(1098) | .419 |
|  |  |  |  |  |  |  |  |  |  |  |
| 4 years | 11 | 2.0 | |  | 14 | 2.5 | |  | 3.55(4) | .470 |
| 5-6 years | 272 | 50.3 | |  | 266 | 47.6 | |  |  |  |
| 7-8 years | 146 | 27.0 | |  | 151 | 27.0 | |  |  |  |
| 9-10 years | 108 | 20.0 | |  | 127 | 22.7 | |  |  |  |
| 11 years | 4 | 0.7 | |  | 1 | 0.2 | |  |  |  |
|  |  |  |  |  |  |  |  |  |  |  |
| **Sex of child** |  |  |  |  |  |  |  |  |  |  |
| male | 244 | 45.2 | |  | 268 | 47.9 | |  | 0.79(1) | .374 |
| female | 296 | 54.8 | |  | 292 | 52.1 | |  |  |  |
|  |  |  |  |  |  |  |  |  |  |  |
| **Nationality of mother** |  |  |  |  |  |  |  |  |  |  |
| German | 395 | 95.9 | |  | 461 | 97.9 | |  | 2.97(1) | .085 |
| other | 17 | 4.1 | |  | 10 | 2.1 | |  |  |  |
| **Nationality of father** |  |  |  |  |  |  |  |  |  |  |
| German | 384 | 97.5 | |  | 445 | 97.8 | |  | 0.11(1) | .744 |
| other | 10 | 2.5 | |  | 10 | 2.2 | |  |  |  |
| **Nationality of child** |  |  |  |  |  |  |  |  |  |  |
| German | 403 | 98.8 | |  | 460 | 98.9 | |  | 0.04(1) | .835 |
| other | 5 | 1.2 | |  | 5 | 1.1 | |  |  |  |
|  |  |  |  |  |  |  |  |  |  |  |
| **Institution** |  |  |  |  |  |  |  |  |  |  |
| kindergarten/preschool | 258 | 50.9 | |  | 280 | 49.9 | |  | 0.10(1) | .750 |
| school | 249 | 49.1 | |  | 281 | 50.1 | |  |  |  |
|  |  |  |  |  |  |  |  |  |  |  |
| **Siblings** |  |  |  |  |  |  |  |  |  |  |
| at least one | 314 | 83.1 | |  | 452 | 80.6 | |  | 0.94(1) | .333 |
| none | 64 | 16.9 | |  | 109 | 19.4 | |  |  |  |
|  |  |  |  |  |  |  |  |  |  |  |
| **Living situation** |  |  |  |  |  |  |  |  |  |  |
| singel parent | 45 | 10.9 | |  | 44 | 9.4 | |  | 1.02(2) | .601 |
| permanent partnership/married | 354 | 85.9 | |  | 407 | 86.6 | |  |  |  |
| other | 13 | 3.2 | |  | 19 | 4.0 | |  |  |  |
|  |  |  |  |  |  |  |  |  |  |  |
| **Houshold net income after tax** |  |  |  |  |  |  |  |  |  |  |
| less than 1000€ | 5 | 1.4 | |  | 4 | 0.9 | |  | 1.04(4) | .904 |
| 1000-2000€ | 39 | 10.6 | |  | 49 | 11.3 | |  |  |  |
| 2000-3000€ | 64 | 17.4 | |  | 68 | 15.7 | |  |  |  |
| 3000-4000€ | 139 | 37.8 | |  | 160 | 37.0 | |  |  |  |
| more than 4000€ | 121 | 32.9 | |  | 151 | 35.0 | |  |  |  |
|  |  |  |  |  |  |  |  |  |  |  |
| **Mother's highest educational qualification** |  |  |  |  |  |  |  |  |  |  |
| without educational qualifications | 1 | 0.2 | |  | 0 | 0.0 | |  | 10.41(5) | .064 |
| lower secondary education | 10 | 2.4 | |  | 12 | 2.6 | |  |  |  |
| secondary school certificate | 116 | 28.2 | |  | 119 | 25.3 | |  |  |  |
| higher education entrance qualification | 93 | 22.6 | |  | 95 | 20.2 | |  |  |  |
| graduate degree | 189 | 46.0 | |  | 231 | 49.0 | |  |  |  |
| other | 2 | 0.5 | |  | 14 | 3.0 | |  |  |  |
|  |  |  |  |  |  |  |  |  |  |  |
| **Father's highest educational qualification** |  |  |  |  |  |  |  |  |  |  |
| without educational qualifications | 2 | 0.5 | |  | 2 | 0.4 | |  | 5.29(5) | .381 |
| lower secondary education | 19 | 4.8 | |  | 19 | 4.2 | |  |  |  |
| secondary school certificate | 126 | 31.8 | |  | 127 | 27.9 | |  |  |  |
| higher education entrance qualification | 67 | 16.9 | |  | 65 | 14.3 | |  |  |  |
| graduate degree | 170 | 42.9 | |  | 231 | 50.8 | |  |  |  |
| other | 12 | 3.0 | |  | 11 | 2.4 | |  |  |  |
|  |  |  |  |  |  |  |  |  |  |  |
| **SDQ (Screening)** |  |  | |  |  |  | |  |  |  |
| **emotional problems scale score**  **[M(SD), t(df)]** | 538 | 1.8(2.0) | |  | 556 | 2.1(2.1) | |  | **-2.03(1091.9)** | **.043** |
|  |  |  |  |  |  |  |  |  |  |  |
| normal | 444 | 82.5 | |  | 442 | 79.5 | |  | 1.76(2) | .416 |
| borderline | 74 | 13.8 | |  | 92 | 16.6 | |  |  |  |
| abnormal | 20 | 3.7 | |  | 22 | 4.0 | |  |  |  |
|  |  |  |  |  |  |  |  |  |  |  |
| **total score [M(SD), t(df)]** | 538 | 6.9(4.7) | |  | 556 | 7.3(4.9) | |  | -1.41(1092) | .159 |
|  |  |  |  |  |  |  |  |  |  |  |
| normal | 488 | 90.7 | |  | 500 | 89.9 | |  | 0.23(2) | .891 |
| borderline | 26 | 4.8 | |  | 28 | 5.0 | |  |  |  |
| abnormal | 24 | 4.5 | |  | 28 | 5.0 | |  |  |  |
|  |  |  |  |  |  |  |  |  |  |  |
| **CES-DC (T0)** |  |  |  |  |  |  |  |  |  |  |
| total score [M(SD), t(df)] | 355 | 7.1(4.9) | |  | 561 | 7.3(5.5) | |  | -0.39(914) | .697 |
|  |  |  |  |  |  |  |  |  |  |  |
| **SCARED (T0)** |  |  |  |  |  |  |  |  |  |  |
| social anxiety scale score [M(SD), t(df)] | 363 | 4.5(3.6) | |  | 561 | 5.1(4.0) | |  | **-2.32(820.8)** | **.021** |
| total score [M(SD), t(df)] | 363 | 12.4(9.4) | |  | 561 | 13.8(10.4) | |  | **-2.15(922)** | **.032** |
| Note. Completer: data on CES and SCARED available at all measurement times; Non-Completer: data on CES or SCARED available at least at one but not all measurement time points; NOR: children who were not recommended to participate in a prevention program because they were assessed as normal; TT: children who participated in the prevention program "Mutig werden mit Til Tiger" (Becoming brave with Til Tiger), i.e. attended at least one session; NoTT: children who were recommended to participate in the Til Tiger program but did not do so; all variables except siblings were assessed at the screening timepoint; if institution information was missing at screening, information from timepoint t0 was used; sibling were assessed at timepoint t0; n: number of cases; M: mean; SD: standard deviation; Chi2: Chi Square Test (for categorical variables); t: (Welch's) t-test (for continuous variables); df: degrees of freedom; bold prints indicate statistical significance, p<.05 | | | | | | | | | | |

| Online Resource 5. Multilevel regression models for anxiety, social anxiety, and depression on the interaction of group and time point without prior multiple imputation. | | | | | | | | | | | | | | | | |
| --- | --- | --- | --- | --- | --- | --- | --- | --- | --- | --- | --- | --- | --- | --- | --- | --- |
|  | SCARED Anxiety | | | |  | SCARED Social Anxiety | | | | |  | | CES-DC Depression | | | |
|  | β | SE | 95%CI | p |  | β | SE | 95%CI | p |  | | β | | SE | 95%CI | p |
| **Model 1: NOR as reference** |  |  |  |  |  |  |  |  |  |  | |  | |  |  |  |
| Measurement Time Point |  |  |  |  |  |  |  |  |  |  | |  | |  |  |  |
| T0 | reference |  |  |  |  |  |  |  |  |  | |  | |  |  |  |
| T1 | 0.05 | 0.09 | -0.12 – 0.22 | .549 |  | 0.04 | 0.08 | -0.13 – 0.20 | .645 |  | | 0.03 | | 0.11 | -0.20 – 0.25 | .819 |
| T2 | 0.13 | 0.17 | -0.20 – 0.46 | .433 |  | 0.12 | 0.17 | -0.21 – 0.45 | .479 |  | | -0.03 | | 0.22 | -0.47 – 0.41 | .895 |
|  |  |  |  |  |  |  |  |  |  |  | |  | |  |  |  |
| Group |  |  |  |  |  |  |  |  |  |  | |  | |  |  |  |
| NOR | reference |  |  |  |  |  |  |  |  |  | |  | |  |  |  |
| TT | **2.23** | **0.12** | **2.01** – **2.46** | **<.001** |  | **1.99** | **0.10** | **1.79** – **2.18** | **<.001** |  | | **1.22** | | **0.12** | **0.98** – **1.46** | **<.001** |
| NoTT | **1.11** | **0.18** | **0.76** – **1.46** | **<.001** |  | **0.78** | **0.16** | **0.47** – **1.09** | **<.001** |  | | **0.55** | | **0.19** | **0.17** – **0.93** | **.005** |
|  |  |  |  |  |  |  |  |  |  |  | |  | |  |  |  |
|  |  |  |  |  |  |  |  |  |  |  | |  | |  |  |  |
| Time Point x Group |  |  |  |  |  |  |  |  |  |  | |  | |  |  |  |
| T1 x TT | **-0.75** | **0.11** | **-0.97** – **-0.52** | **<.001** |  | **-0.49** | **0.09** | **-0.67** – **-0.30** | **<.001** |  | | **-0.51** | | **0.14** | **-0.79** – **-0.23** | **<.001** |
| T1 x NoTT | -0.03 | 0.16 | -0.34 – 0.29 | .864 |  | 0.04 | 0.17 | -0.29 – 0.36 | .823 |  | | 0.20 | | 0.23 | -0.26 – 0.66 | .394 |
| T2 x TT | **-0.97** | **0.12** | **-1.20** – **-0.73** | **<.001** |  | **-0.71** | **0.11** | **-0.92** – **-0.49** | **<.001** |  | | **-0.46** | | **0.14** | **-0.74** – **-0.18** | **<.001** |
| T2 x NoTT | -0.06 | 0.18 | -0.40 – 0.29 | .743 |  | -0.13 | 0.14 | -0.41 – 0.14 | .339 |  | | 0.28 | | 0.27 | -0.25 – 0.82 | .304 |
|  |  |  |  |  |  |  |  |  |  |  | |  | |  |  |  |
| age of child | **0.03** | **0.01** | **0.00** – **0.06** | **.032** |  | -0.01 | 0.01 | -0.04 – 0.01 | .365 |  | | **0.04** | | **0.01** | **0.01** – **0.07** | **<.001** |
| sex of child | -0.07 | 0.06 | -0.18 – 0.05 | .245 |  | -0.08 | 0.06 | -0.19 – 0.04 | .181 |  | | 0.05 | | 0.05 | -0.06 – 0.15 | .397 |
| time gap in days | 0.00 | 0.00 | 0.00 – 0.00 | .309 |  | 0.00 | 0.00 | 0.00 – 0.00 | .405 |  | | 0.00 | | 0.00 | 0.00 – 0.00 | .670 |
| intercept | **-0.47** | **0.11** | **-0.69** – **-0.25** | **<.001** |  | -0.13 | 0.11 | -0.35 – 0.09 | .241 |  | | **-0.54** | | **0.11** | **-0.75** – **-0.33** | **<.001** |
|  |  |  |  |  |  |  |  |  |  |  | |  | |  |  |  |
| **Model 2: NotTT as reference** |  |  |  |  |  |  |  |  |  |  | |  | |  |  |  |
| Measurement Time Point |  |  |  |  |  |  |  |  |  |  | |  | |  |  |  |
| T0 | reference |  |  |  |  |  |  |  |  |  | |  | |  |  |  |
| T1 | 0.02 | 0.17 | -0.30 – 0.35 | .887 |  | 0.08 | 0.17 | -0.26 – 0.41 | .659 |  | | 0.23 | | 0.24 | -0.25 – 0.70 | .351 |
| T2 | 0.07 | 0.22 | -0.35 – 0.50 | .728 |  | -0.02 | 0.18 | -0.37 – 0.34 | .930 |  | | 0.25 | | 0.33 | -0.40 – 0.90 | .451 |
|  |  |  |  |  |  |  |  |  |  |  | |  | |  |  |  |
| Group |  |  |  |  |  |  |  |  |  |  | |  | |  |  |  |
| NoTT | reference |  |  |  |  |  |  |  |  |  | |  | |  |  |  |
| NOR | **-1.11** | **0.18** | **-1.46** – **-0.76** | **<.001** |  | **-0.78** | **0.16** | **-1.09** – **-0.47** | **<.001** |  | | **-0.55** | | **0.19** | **-0.93** – **-0.17** | **.005** |
| TT | **1.13** | **0.21** | **0.72** – **1.53** | **<.001** |  | **1.21** | **0.18** | **0.86** – **1.55** | **<.001** |  | | **0.67** | | **0.22** | **0.25** – **1.10** | **<.001** |
|  |  |  |  |  |  |  |  |  |  |  | |  | |  |  |  |
|  |  |  |  |  |  |  |  |  |  |  | |  | |  |  |  |
| Time Point x Group |  |  |  |  |  |  |  |  |  |  | |  | |  |  |  |
| T1 x NOR | 0.03 | 0.16 | -0.29 – 0.34 | .864 |  | -0.04 | 0.17 | -0.36 – 0.29 | .823 |  | | -0.20 | | 0.23 | -0.66 – 0.26 | .394 |
| T1 x TT | **-0.72** | **0.19** | **-1.09** – **-0.35** | **<.001** |  | **-0.52** | **0.18** | **-0.88** – **-0.16** | **<.001** |  | | **-0.71** | | **0.26** | **-1.23** – **-0.19** | **.007** |
| T2 x NOR | 0.06 | 0.18 | -0.29 – 0.40 | .743 |  | 0.13 | 0.14 | -0.14 – 0.41 | .339 |  | | -0.28 | | 0.27 | -0.82 – 0.25 | .304 |
| T2 x TT | **-0.91** | **0.20** | **-1.31** – **-0.51** | **<.001** |  | **-0.58** | **0.17** | **-0.90** – **-0.25** | **<.001** |  | | **-0.74** | | **0.30** | **-1.33** – **-0.14** | **.015** |
|  |  |  |  |  |  |  |  |  |  |  | |  | |  |  |  |
| age of child | **0.03** | **0.01** | **0.00** – **0.06** | **.032** |  | -0.01 | 0.01 | -0.04 – 0.01 | .365 |  | | **0.04** | | **0.01** | **0.01** – **0.07** | **<.001** |
| sex of child | -0.07 | 0.06 | -0.18 – 0.05 | .245 |  | -0.08 | 0.06 | -0.19 – 0.04 | .181 |  | | 0.05 | | 0.05 | -0.06 – 0.15 | .397 |
| time gap in days | 0.00 | 0.00 | 0.00 – 0.00 | .309 |  | 0.00 | 0.00 | 0.00 – 0.00 | .405 |  | | 0.00 | | 0.00 | 0.00 – 0.00 | .670 |
| intercept | **0.63** | **0.21** | **0.23** – **1.04** | **<.001** |  | **0.65** | **0.19** | **0.27** – **1.03** | **<.001** |  | | 0.01 | | 0.22 | -0.43 – 0.45 | .974 |
| Note. T0, measurement time point before program participation; T1, first measurement time point after program participation, (approximately 3-5 months after T0); T2, second measurement time point after program participation, (approximately 9-11 months after T0); NOR: children who were not recommended to participate in a prevention program because they were assessed as normal; TT: children who participated in the prevention program "Mutig werden mit Til Tiger" (Becoming brave with Til Tiger), i.e. attended at least one session; NoTT: children who were recommended to participate in the Til Tiger program but did not do so; group and time point were coded as a dummy, using T0 and NOR or NoTT as the reference; all models were adjusted for age of child, sex of child (0 = female, 1 = male) and time in days between measurements (time gap); Due to 4 missing values in the sex variable, the n is reduced for analyses without prior multiple imputation. CI, confidence interval; SE, standard error; bold indicates statistical significance, p<.05. | | | | | | | | | | | | | | | | |

| Online Resource 6. Multilevel regression models for anxiety, social anxiety, and depression on the interaction of group, sex of child, and time point using multiply imputed data. | | | | | | | | | | | | | | | | | | | | | | | |
| --- | --- | --- | --- | --- | --- | --- | --- | --- | --- | --- | --- | --- | --- | --- | --- | --- | --- | --- | --- | --- | --- | --- | --- |
|  | SCARED Anxiety | | | | | | |  | SCARED Social Anxiety | | | | | | |  | CES-DC Depression | | | | | | |
|  | β |  | SE |  | 95%CI | | p |  | β |  | SE |  | 95%CI | | p |  | β |  | SE |  | 95%CI | | p |
| **Measurement Time Point** |  |  |  |  |  |  |  |  |  |  |  |  |  |  |  |  |  |  |  |  |  |  |  |
| T0 | reference |  |  |  |  |  |  |  |  |  |  |  |  |  |  |  |  |  |  |  |  |  |  |
| T1 | 0.01 |  | 0.09 |  | -0.16 – 0.18 | | .883 |  | -0.01 |  | 0.09 |  | -0.18 – 0.16 | | .912 |  | 0.08 |  | 0.12 |  | -0.15 – 0.31 | | .493 |
| T2 | 0.09 |  | 0.17 |  | -0.25 – 0.43 | | .623 |  | 0.09 |  | 0.17 |  | -0.24 – 0.43 | | .574 |  | 0.01 |  | 0.22 |  | -0.42 – 0.44 | | .966 |
|  |  |  |  |  |  |  |  |  |  |  |  |  |  |  |  |  |  |  |  |  |  |  |  |
| **Group** |  |  |  |  |  |  |  |  |  |  |  |  |  |  |  |  |  |  |  |  |  |  |  |
| NOR | reference |  |  |  |  |  |  |  |  |  |  |  |  |  |  |  |  |  |  |  |  |  |  |
| TT | **2.13** |  | **0.17** |  | **1.80** – **2.46** | | **<.001** |  | **1.88** |  | **0.15** |  | **1.59** – **2.18** | | **<.001** |  | **1.21** |  | **0.19** |  | **0.82** – **1.59** | | **<.001** |
| NoTT | **1.13** |  | **0.21** |  | **0.73** – **1.54** | | **<.001** |  | **0.74** |  | **0.20** |  | **0.34** – **1.13** | | **<.001** |  | **0.55** |  | **0.24** |  | **0.07** – **1.03** | | **.025** |
|  |  |  |  |  |  |  |  |  |  |  |  |  |  |  |  |  |  |  |  |  |  |  |  |
| **Time Point x Group** |  |  |  |  |  |  |  |  |  |  |  |  |  |  |  |  |  |  |  |  |  |  |  |
| T1 x TT | **-0.72** |  | **0.16** |  | **-1.02** – **-0.41** | | **<.001** |  | **-0.35** |  | **0.12** |  | **-0.58** – **-0.11** | | **.004** |  | **-0.70** |  | **0.21** |  | **-1.12** – **-0.29** | | **.001** |
| T1 x NoTT | -0.26 |  | 0.20 |  | -0.65 – 0.13 | | .195 |  | -0.08 |  | 0.23 |  | -0.53 – 0.36 | | .716 |  | 0.26 |  | 0.35 |  | -0.42 – 0.93 | | .461 |
| T2 x TT | **-0.90** |  | **0.16** |  | **-1.22** – **-0.59** | | **<.001** |  | **-0.68** |  | **0.14** |  | **-0.96** – **-0.40** | | **<.001** |  | **-0.53** |  | **0.18** |  | **-0.90** – **-0.17** | | **.004** |
| T2 x NoTT | 0.00 |  | 0.24 |  | -0.47 – 0.47 | | .989 |  | -0.14 |  | 0.19 |  | -0.51 – 0.23 | | .459 |  | 0.46 |  | 0.38 |  | -0.29 – 1.22 | | .230 |
|  |  |  |  |  |  |  |  |  |  |  |  |  |  |  |  |  |  |  |  |  |  |  |  |
| **Sex of child** |  |  |  |  |  |  |  |  |  |  |  |  |  |  |  |  |  |  |  |  |  |  |  |
| female | reference |  |  |  |  |  |  |  |  |  |  |  |  |  |  |  |  |  |  |  |  |  |  |
| male | -0.11 |  | 0.07 |  | -0.24 – 0.02 | | .098 |  | -0.12 |  | 0.07 |  | -0.26 – 0.02 | | .090 |  | 0.06 |  | 0.07 |  | -0.07 – 0.19 | | .345 |
|  |  |  |  |  |  |  |  |  |  |  |  |  |  |  |  |  |  |  |  |  |  |  |  |
| **Time Point x Sex of child** |  |  |  |  |  |  |  |  |  |  |  |  |  |  |  |  |  |  |  |  |  |  |  |
| T1 x male | 0.02 |  | 0.06 |  | -0.09 – 0.13 | | .727 |  | 0.07 |  | 0.06 |  | -0.05 – 0.18 | | .281 |  | -0.11 |  | 0.08 |  | -0.25 – 0.04 | | .162 |
| T2 x male | -0.01 |  | 0.07 |  | -0.14 – 0.12 | | .896 |  | -0.03 |  | 0.07 |  | -0.16 – 0.10 | | .672 |  | -0.04 |  | 0.08 |  | -0.20 – 0.13 | | .656 |
|  |  |  |  |  |  |  |  |  |  |  |  |  |  |  |  |  |  |  |  |  |  |  |  |
| **Group x Sex of child** |  |  |  |  |  |  |  |  |  |  |  |  |  |  |  |  |  |  |  |  |  |  |  |
| TT x male | 0.26 |  | 0.23 |  | -0.18 – 0.71 | | .246 |  | 0.24 |  | 0.20 |  | -0.14 – 0.63 | | .215 |  | 0.05 |  | 0.24 |  | -0.42 – 0.52 | | .833 |
| NoTT x male | -0.09 |  | 0.38 |  | -0.84 – 0.66 | | .821 |  | 0.09 |  | 0.32 |  | -0.54 – 0.72 | | .779 |  | -0.08 |  | 0.38 |  | -0.83 – 0.66 | | .826 |
|  |  |  |  |  |  |  |  |  |  |  |  |  |  |  | |  |  |  |  |  |  |  |  |
| **Time Point x Group x Sex of child** |  |  |  |  |  |  |  |  |  |  |  |  |  |  |  |  |  |  |  |  |  |  |  |
| T1 x TT x male | -0.07 |  | 0.22 |  | -0.49 – 0.35 | | .747 |  | -0.28 |  | 0.17 |  | -0.62 – 0.07 | | .113 |  | 0.37 |  | 0.27 |  | -0.17 – 0.90 | | .179 |
| T1 x NoTT x male | 0.61 |  | 0.32 |  | -0.01 – 1.23 | | .054 |  | 0.31 |  | 0.31 |  | -0.29 – 0.92 | | .305 |  | -0.12 |  | 0.42 |  | -0.95 – 0.70 | | .768 |
| T2 x TT x male | -0.13 |  | 0.24 |  | -0.60 – 0.33 | | .569 |  | -0.06 |  | 0.21 |  | -0.48 – 0.36 | | .785 |  | 0.16 |  | 0.29 |  | -0.40 – 0.72 | | .574 |
| T2 x NoTT x male | 0.06 |  | 0.34 |  | -0.60 – 0.72 | | .862 |  | 0.09 |  | 0.27 |  | -0.45 – 0.62 | | .753 |  | -0.26 |  | 0.54 |  | -1.32 – 0.79 | | .624 |
|  |  |  |  |  |  |  |  |  |  |  |  |  |  |  |  |  |  |  |  |  |  |  |  |
| age of child | **0.03** |  | **0.02** |  | 1. **0.06** | | **.033** |  | -0.01 |  | 0.01 |  | -0.04 – 0.01 | | .324 |  | **0.04** |  | **0.01** |  | **0.01 – 0.07** | | **.004** |
| time gap in days | 0.00 |  | 0.00 |  | 0.00 – 0.00 | | .487 |  | 0.00 |  | 0.00 |  | 0.00 – 0.00 | | .568 |  | 0.00 |  | 0.00 |  | 0.00 – 0.00 | | .709 |
| intercept | **-0.45** |  | **0.12** |  | **-0.68** – **-0.22** | | **<.001** |  | -0.10 |  | 0.11 |  | -0.32 – 0.13 | | .393 |  | **-0.54** |  | **0.11** |  | **-0.76** – **-0.33** | | **<.001** |

*Note.* All models were run with multiply imputed data. T0, measurement time point before program participation; T1, first measurement time point after program participation, (approximately 3-5 months after T0); T2, second measurement time point after program participation, (approximately 9-11 months after T0); TT: children who participated in the prevention program "Mutig werden mit Til Tiger" (Becoming brave with Til Tiger), i.e. attended at least one session; NoTT: children who were recommended to participate in the Til Tiger program but did not do so; group, sex, and time point were coded as a dummy, using NOR, female, and T0 as the reference; all models were adjusted for age of child and time in days between measurements (time gap); CI, confidence interval; SE, standard error; bold indicates statistical significance, p<.05.

| Online Resource 7. Multilevel regression models for anxiety, social anxiety, and depression on the interaction of group, age of child, and time point using multiply imputed data. | | | | | | | | | | | | | | | | | | | | | | | |
| --- | --- | --- | --- | --- | --- | --- | --- | --- | --- | --- | --- | --- | --- | --- | --- | --- | --- | --- | --- | --- | --- | --- | --- |
|  | SCARED Anxiety | | | | | | |  | SCARED Social Anxiety | | | | | | |  | CES-DC Depression | | | | | | |
|  | β |  | SE |  | 95%CI | | p |  | β |  | SE |  | 95%CI | | p |  | β |  | SE |  | 95%CI | | p |
| **Measurement Time Point** |  |  |  |  |  |  |  |  |  |  |  |  |  |  |  |  |  |  |  |  |  |  |  |
| T0 | reference |  |  |  |  |  |  |  |  |  |  |  |  |  |  |  |  |  |  |  |  |  |  |
| T1 | 0.01 |  | 0.09 |  | -0.16 – 0.18 | | .889 |  | 0.04 |  | 0.09 |  | -0.13 – 0.21 | | .674 |  | -0.03 |  | 0.12 |  | -0.26 – 0.20 | | .769 |
| T2 | 0.14 |  | 0.17 |  | -0.19 – 0.47 | | .400 |  | 0.09 |  | 0.17 |  | -0.24 – 0.42 | | .580 |  | -0.05 |  | 0.23 |  | -0.50 – 0.40 | | .816 |
|  |  |  |  |  |  |  |  |  |  |  |  |  |  |  |  |  |  |  |  |  |  |  |  |
| **Group** |  |  |  |  |  |  |  |  |  |  |  |  |  |  |  |  |  |  |  |  |  |  |  |
| NOR | reference |  |  |  |  |  |  |  |  |  |  |  |  |  |  |  |  |  |  |  |  |  |  |
| TT | **2.29** |  | **0.16** |  | **1.97** – **2.61** | | **<.001** |  | **2.02** |  | **0.14** |  | **1.75** – **2.30** | | **<.001** |  | **1.35** |  | **0.16** |  | **1.04** – **1.66** | | **<.001** |
| NoTT | **0.98** |  | **0.24** |  | **0.51** – **1.46** | | **<.001** |  | **0.79** |  | **0.22** |  | **0.35** – **1.23** | | **<.001** |  | 0.52 |  | 0.27 |  | -0.01 – 1.04 | | .052 |
|  |  |  |  |  |  |  |  |  |  |  |  |  |  |  |  |  |  |  |  |  |  |  |  |
| **Time Point x Group** |  |  |  |  |  |  |  |  |  |  |  |  |  |  |  |  |  |  |  |  |  |  |  |
| T1 x TT | **-0.74** |  | **0.18** |  | **-1.08** – **-0.40** | | **<.001** |  | **-0.55** |  | **0.14** |  | **-0.83** – **-0.27** | | **<.001** |  | -0.45 |  | 0.23 |  | -0.91 – 0.00 | | .052 |
| T1 x NoTT | 0.05 |  | 0.23 |  | -0.40 – 0.51 | | .823 |  | 0.10 |  | 0.20 |  | -0.29 – 0.49 | | .619 |  | 0.11 |  | 0.32 |  | -0.51 – 0.74 | | .721 |
| T2 x TT | **-1.03** |  | **0.17** |  | **-1.37** – **-0.69** | | **<.001** |  | **-0.81** |  | **0.18** |  | **-1.17** – **-0.45** | | **<.001** |  | **-0.49** |  | **0.24** |  | **-0.95** – **-0.03** | | **.038** |
| T2 x NoTT | -0.07 |  | 0.25 |  | -0.55 – 0.42 | | .791 |  | -0.24 |  | 0.20 |  | -0.63 – 0.15 | | .229 |  | -0.06 |  | 0.29 |  | -0.63 – 0.51 | | .836 |
|  |  |  |  |  |  |  |  |  |  |  |  |  |  |  |  |  |  |  |  |  |  |  |  |
| **Age of child** |  |  |  |  |  |  |  |  |  |  |  |  |  |  |  |  |  |  |  |  |  |  |  |
| younger | reference |  |  |  |  |  |  |  |  |  |  |  |  |  |  |  |  |  |  |  |  |  |  |
| older | 0.11 |  | 0.07 |  | -0.03 – 0.24 | | .124 |  | -0.07 |  | 0.07 |  | -0.20 – 0.07 | | .347 |  | 0.07 |  | 0.07 |  | -0.06 – 0.21 | | .266 |
|  |  |  |  |  |  |  |  |  |  |  |  |  |  |  |  |  |  |  |  |  |  |  |  |
| **Time Point x Age of child** |  |  |  |  |  |  |  |  |  |  |  |  |  |  |  |  |  |  |  |  |  |  |  |
| T1 x older | 0.03 |  | 0.06 |  | -0.08 – 0.14 | | .586 |  | -0.03 |  | 0.06 |  | -0.14 – 0.09 | | .643 |  | 0.11 |  | 0.08 |  | -0.04 – 0.26 | | .151 |
| T2 x older | -0.07 |  | 0.07 |  | -0.20 – 0.05 | | .261 |  | -0.03 |  | 0.07 |  | -0.16 – 0.10 | | .666 |  | 0.08 |  | 0.08 |  | -0.08 – 0.24 | | .316 |
|  |  |  |  |  |  |  |  |  |  |  |  |  |  |  |  |  |  |  |  |  |  |  |  |
| **Group x Age of child** |  |  |  |  |  |  |  |  |  |  |  |  |  |  |  |  |  |  |  |  |  |  |  |
| TT x older | -0.04 |  | 0.24 |  | -0.51 – 0.43 | | .864 |  | -0.03 |  | 0.20 |  | -0.43 – 0.37 | | .897 |  | -0.26 |  | 0.22 |  | -0.68 – 0.17 | | .233 |
| NoTT x older | 0.28 |  | 0.33 |  | -0.37 – 0.92 | | .402 |  | -0.03 |  | 0.30 |  | -0.61 – 0.56 | | .926 |  | 0.00 |  | 0.38 |  | -0.74 – 0.74 | | .997 |
|  |  |  |  |  |  |  |  |  |  |  |  |  |  |  |  |  |  |  |  |  |  |  |  |
| **Time Point x Group x Age of child** |  |  |  |  |  |  |  |  |  |  |  |  |  |  |  |  |  |  |  |  |  |  |  |
| T1 x TT x older | -0.03 |  | 0.22 |  | -0.46 – 0.40 | | .889 |  | 0.12 |  | 0.18 |  | -0.22 – 0.47 | | .484 |  | -0.10 |  | 0.27 |  | -0.64 – 0.44 | | .716 |
| T1 x NoTT x older | -0.17 |  | 0.32 |  | -0.79 – 0.45 | | .597 |  | -0.15 |  | 0.34 |  | -0.81 – 0.51 | | .648 |  | 0.22 |  | 0.47 |  | -0.69 – 1.14 | | .631 |
| T2 x TT x older | 0.09 |  | 0.23 |  | -0.36 – 0.54 | | .689 |  | 0.18 |  | 0.22 |  | -0.25 – 0.61 | | .410 |  | 0.09 |  | 0.31 |  | -0.52 – 0.69 | | .784 |
| T2 x NoTT x older | 0.12 |  | 0.36 |  | -0.58 – 0.82 | | .743 |  | 0.27 |  | 0.28 |  | -0.27 – 0.82 | | .329 |  | 0.94 |  | 0.60 |  | -0.23 – 2.12 | | .114 |
|  |  |  |  |  |  |  |  |  |  |  |  |  |  |  |  |  |  |  |  |  |  |  |  |
| sex of child | -0.07 |  | 0.06 |  | -0.19 – 0.05 | | .232 |  | -0.08 |  | 0.06 |  | -0.19 – 0.04 | | .183 |  | 0.04 |  | 0.06 |  | -0.07 – 0.15 | | .503 |
| time gap in days | 0.00 |  | 0.00 |  | 0.00 – 0.00 | | .512 |  | 0.00 |  | 0.00 |  | 0.00 – 0.00 | | .519 |  | 0.00 |  | 0.00 |  | 0.00 – 0.00 | | .585 |
| intercept | **-0.30** |  | **0.06** |  | **-0.42** – **-0.18** | | **<.001** |  | **-0.18** |  | **0.06** |  | **-0.31** – **-0.05** | | **.005** |  | **-0.30** |  | **0.06** |  | **-0.43** – **-0.18** | | **<.001** |

*Note.* All models were run with multiply imputed data. T0, measurement time point before program participation; T1, first measurement time point after program participation, (approximately 3-5 months after T0); T2, second measurement time point after program participation, (approximately 9-11 months after T0); TT: children who participated in the prevention program "Mutig werden mit Til Tiger" (Becoming brave with Til Tiger), i.e. attended at least one session; NoTT: children who were recommended to participate in the Til Tiger program but did not do so; group, age (0 = 6 years or younger, 1 = 7 years or older), and time point were coded as a dummy, using NOR, younger, and T0 as the reference; all models were adjusted for sex of child (0 = female, 1 = male) and time in days between measurements (time gap); CI, confidence interval; SE, standard error; bold indicates statistical significance, p<.05.

| Online Resource 8. Multilevel regression models for anxiety, social anxiety, and depression on the interaction of group, median group, and time point using multiply imputed data. | | | | | | | | | | | | | | |
| --- | --- | --- | --- | --- | --- | --- | --- | --- | --- | --- | --- | --- | --- | --- |
|  | SCARED Anxiety | | | |  | SCARED Social Anxiety | | | |  | CES-DC Depression | | | |
|  | β | SE | 95%CI | p |  | β | SE | 95%CI | p |  | β | SE | 95%CI | p |
| **Measurement Time Point** |  |  |  |  |  |  |  |  |  |  |  |  |  |  |
| T0 | reference |  |  |  |  |  |  |  |  |  |  |  |  |  |
| T1 | -0.24 | 0.15 | -0.53 – 0.05 | .108 |  | **-0.29** | **0.13** | **-0.55** – **-0.02** | **.033** |  | 0.15 | 0.16 | -0.16 – 0.46 | .347 |
| T2 | -0.47 | 0.27 | -1.00 – 0.07 | .089 |  | -0.45 | 0.25 | -0.95 – 0.04 | .072 |  | 0.15 | 0.36 | -0.55 – 0.85 | .671 |
| **Group** |  |  |  |  |  |  |  |  |  |  |  |  |  |  |
| TT | reference |  |  |  |  |  |  |  |  |  |  |  |  |  |
| NoTT | **-1.15** | **0.14** | **-1.42** – **-0.89** | **<.001** |  | **-1.39** | **0.16** | **-1.71** – **-1.07** | **<.001** |  | **-0.52** | **0.13** | **-0.76** – **-0.27** | **<.001** |
|  |  |  |  |  |  |  |  |  |  |  |  |  |  |  |
| **Time Point x Group** |  |  |  |  |  |  |  |  |  |  |  |  |  |  |
| T1 x NoTT | 0.31 | 0.22 | -0.13 – 0.74 | .165 |  | **0.62** | **0.21** | **0.21** – **1.04** | **.003** |  | **0.63** | **0.26** | **0.12** – **1.15** | **.016** |
| T2 x NoTT | **0.79** | **0.22** | **0.36** – **1.21** | **<.001** |  | **0.56** | **0.19** | **0.19** – **0.94** | **.003** |  | **0.90** | **0.31** | **0.30** – **1.50** | **.003** |
| **Median Group** |  |  |  |  |  |  |  |  |  |  |  |  |  |  |
| low | reference |  |  |  |  |  |  |  |  |  |  |  |  |  |
| high | **2.03** | **0.14** | **1.76** – **2.30** | **<.001** |  | **1.65** | **0.11** | **1.44** – **1.86** | **<.001** |  | **2.09** | **0.15** | **1.79** – **2.39** | **<.001** |
| **Time Point x Median Group** |  |  |  |  |  |  |  |  |  |  |  |  |  |  |
| T1 x high |  | **0.19** | **-1.46** – **-0.70** | **<.001** |  | **-0.42** | **0.16** | **-0.74** – **-0.10** | **.009** |  | **-1.24** | **0.25** | **-1.73** – **-0.75** | **<.001** |
| T2 x high | **-0.99** | **0.21** | **-1.41** – **-0.57** | **<.001** |  | -0.35 | 0.21 | -0.76 – 0.06 | .098 |  | **-1.04** | **0.26** | **-1.55** – **-0.52** | **<.001** |
| **Group x Mediangroup** |  |  |  |  |  |  |  |  |  |  |  |  |  |  |
| NoTT x high | 0.08 | 0.27 | -0.44 – 0.60 | .763 |  | 0.20 | 0.22 | -0.24 – 0.63 | .382 |  | -0.17 | 0.33 | -0.82 – 0.48 | .605 |
| **Time Point x Group x Median Group** |  |  |  |  |  |  |  |  |  |  |  |  |  |  |
| T1 x NoTT x high | **0.80** | **0.38** | **0.05** – **1.54** | **.037** |  | -0.19 | 0.36 | -0.89 – 0.51 | .600 |  | -0.08 | 0.57 | -1.20 – 1.04 | .885 |
| T2 x NoTT x high | 0.34 | 0.42 | -0.49 – 1.16 | .423 |  | 0.11 | 0.34 | -0.56 – 0.78 | .737 |  | -0.33 | 0.65 | -1.62 – 0.96 | .614 |
|  |  |  |  |  |  |  |  |  |  |  |  |  |  |  |
| age of child | 0.03 | 0.04 | -0.04 – 0.10 | .371 |  | -0.02 | 0.03 | -0.07 – 0.04 | .595 |  | 0.04 | 0.03 | -0.02 – 0.10 | .179 |
| sex of child | 0.07 | 0.13 | -0.18 – 0.33 | .585 |  | 0.04 | 0.11 | -0.18 – 0.27 | .699 |  | 0.00 | 0.13 | -0.26 – 0.25 | .994 |
| time gap in days | 0.00 | 0.00 | 0.00 – 0.00 | .820 |  | 0.00 | 0.00 | 0.00 – 0.00 | .601 |  | 0.00 | 0.00 | 0.00 – 0.00 | .853 |
| intercept | **0.76** | **0.26** | **0.25** – **1.26** | **.003** |  | **1.18** | **0.25** | **0.69** – **1.68** | **<.001** |  | -0.23 | 0.27 | -0.76 – 0.30 | .392 |
| Note. All models were run with multiply imputed data. T0, measurement time point before program participation; T1, first measurement time point after program participation, (approximately 3-5 months after T0); T2, second measurement time point after program participation, (approximately 9-11 months after T0); TT: children who participated in the prevention program "Mutig werden mit Til Tiger" (Becoming brave with Til Tiger), i.e. attended at least one session; NoTT: children who were recommended to participate in the Til Tiger program but did not do so; mediangroup: based on the group-specific median at T0, children were grouped into either the high (> median) or low (<= median) mediangroup; group, mediangroup, and time point were coded as a dummy, using TT, low, and T0 as the reference;all models were adjusted for age of child, sex of child (0 = female, 1 = male) and time in days between measurements (time gap); CI, confidence interval; SE, standard error; bold indicates statistical significance, p<.05. | | | | | | | | | | | | | | |
